# Novel, computational IgE‐clustering in a population‐based cross‐sectional study: Mapping the allergy burden

**DOI:** 10.1002/clt2.12292

**Published:** 2023-09-05

**Authors:** Rebecca Czolk, Maria Ruiz‐Castell, Oliver Hunewald, Naphisabet Wanniang, Gwenaëlle Le Coroller, Christiane Hilger, Michel Vaillant, Guy Fagherazzi, Françoise Morel‐Codreanu, Markus Ollert, Annette Kuehn

**Affiliations:** ^1^ Department of Infection and Immunity Luxembourg Institute of Health Esch‐sur‐Alzette Luxembourg; ^2^ Faculty of Science, Technology and Medicine University of Luxembourg Esch‐sur‐Alzette Luxembourg; ^3^ Epidemiology and Public Health Research Unit Department of Precision Health Luxembourg Institute of Health Strassen Luxembourg; ^4^ Competence Center for Methodology and Statistics Translational Medicine Operations Hub Luxembourg Institute of Health Strassen Luxembourg; ^5^ Department of Allergology and Immunology Centre Hospitalier de Luxembourg‐Kanner Klinik Luxembourg Luxembourg; ^6^ Department of Dermatology and Allergy Center Odense Research Center for Anaphylaxis Odense University Hospital University of Southern Denmark Odense Denmark

**Keywords:** allergy burden, European Health Examination Survey, multiplex IgE‐profiles, population‐based cross‐sectional study

## Abstract

**Background:**

Even though the prevalence of allergies is increasing, population‐based data are still scarce. As a read‐out for chronic inflammatory information, new methods are needed to integrate individual biological measurements and lifestyle parameters to mitigate the consequences and costs of allergic burden for society.

**Methods:**

More than 480.000 data points were collected from 1462 Luxembourg adults during the representative, cross‐sectional European Health Examination Survey, spanning health and lifestyle reports. Deep IgE‐profiles based on unsupervised clustering were correlated with data of the health survey.

**Findings:**

42.6% of the participants reported a physician‐diagnosed allergy and 44% were found to be IgE‐positive to at least one allergen or extract. The main sensitization sources were tree pollens followed by grass pollens and mites (52.4%, 51.8% and 40.3% of sensitized participants respectively), suggesting seasonal as well as perennial burden. The youngest group of participants (25–34 years old) showed the highest burden of sensitization, with 18.2% of them having IgE to 10 or more allergen groups. Unsupervised clustering revealed that the biggest cluster of 24.4% of participants was also the one with the highest medical need, marked by their multi‐sensitization to respiratory sources.

**Interpretation:**

Our novel approach to analyzing large biosample datasets together with health information allows the measurement of the chronic inflammatory disease burden in the general population and led to the identification of the most vulnerable groups in need of better medical care.

## 1 INTRODUCTION

Over the past decades, allergies have consistently increased in prevalence in western countries, expected to affect 50% of the population by 2050.^1^ As a chronic inflammatory disease, they not only give rise to a decreased quality of life for the inflicted but also long‐lasting health impairments.^2^ An atopic phenotype may lead to further comorbidities due to a prematurely aged immune system.^3^, ^4^ Healthcare systems are facing ever‐growing direct costs related to medications and hospitalizations, while national economies are suffering due to indirect costs of allergies, that is, absence at work and loss of productivity.^5^ Allergy development and progression are a consequence of a multitude of complex interwoven factors, such as the genetics of an individual, their living conditions, and allergen exposure during life.^6^ The external exposome is dependent on socioeconomic status, cultural customs and geographical location. Current methods for measuring specific immunoglobulin E (sIgE) as a clinical parameter are not allowing a definitive diagnosis. IgE‐positivity confers allergic sensitization, but so far its assessment has not yet led to a reduction of the “allergy wave,” indicating a need for novel approaches. Although technological advancements allow for new ways of self‐tracking of symptoms and their intensity by patients and therefore prevalence assessment, population‐based studies are still necessary to better understand the specific needs of a community in a given economic and geographical setting.^7^, ^8^ Integrative data analysis of population‐based data may, in the long term, help to estimate and reduce the consequences and costs of allergic burden in societies.

In this study, we demonstrated a new method to analyze large datasets in an allergy context. The European Health Examination Survey is a standardized survey to measure health and lifestyle attributes in European countries.^9^ We used this well‐established cross‐sectional population‐based health survey cohort for Luxembourg^10^ (EHES‐LUX) together with deep personal sIgE profiles and an unsupervised computational approach to build an interconnected picture of allergy in the heart of Western Europe. This novel method allowed us to measure the burden of chronic inflammatory disease in a population. We deeply characterized the Luxembourg population on sensitization and medical burden, identifying groups of highest medical need.

## 2 METHODS

The following are brief descriptions of the methods used in this study. For further information and references, please see the supplementary methods.

### 2.1 Study cohort

Adult participants were included from a representative population‐based cohort established earlier in the frame of the European Health Examination Survey in Luxembourg (EHES‐LUX; *N* = 1529 participants; median age 45; range 26–65 years; Table E2). Questionnaire data from health, lifestyle and environment as well as medical examination data were used, resulting in a total of 165 variables (Table E3). Sera samples available from 1462 participants were applied for IgE‐testing (Figure E1). Statistical analysis ensured that no bias was introduced by biosample availability.

### 2.2 IgE screening and IgE multiplex profiling

EHES‐LUX participants who reported physician‐diagnosed allergies (hereafter called “diagnosed group or diagnosed participants”) were tested in IgE multiplex profiling. Serum IgE‐typing was conducted with 298 allergen(s)/extracts using the Alex2 assay (MacroArrayDX, Wien, Austria; cut‐off for positivity: 0.3 kU_A_/L) at the Luxembourg Institute of Health. For participants who did not report physician‐diagnosed allergies, sera were analyzed with the ImmunoCAP Phadiatop SX1 (Phadia Thermo Fisher Scientific; cut‐off for positivity: 0.35 kU_A_/L) for sIgE‐screening to 8 respiratory allergen sources. The resulting positive samples were also subjected to IgE multiplex profiling (Figure E1).

### 2.3 Unsupervised clustering of participants based on their IgE‐profiles

Macroarray‐derived established sensitization patterns of 298 sIgE values for each sensitized participant were analyzed using R (version 4.1.2) and R Studio (version 2022.02.03+492). Total IgE values were excluded from the unsupervised clustering as quantification using the Alex2 assay is semi‐quantitative only (range of 20.00–2500.00 kU/L). For each established patient cluster, total IgE were then summarized. Data normalization, clustering, and Uniform Manifold Approximation and Projection visualization were performed using the Seurat package (version 4.0.6). Default settings were adjusted to accommodate the lower read depth and dimensions. Log‐normalization was performed using a scale factor of 1000 and the “FindVariableFeatures” function was used with “nfeatures” set to 300. Data scaling and principal component analysis were carried out to build a Nearest‐neighbor graph (“FindNearestNeighbor”). The function “FindCluster” with a resolution of 0.5 resulted in a total of 7 clusters.

### 2.4 Statistical analysis and data visualization

Data analysis was performed in R studio. Descriptive statistics were calculated using the R base functions. The package tidyverse was used for data structuring and cleaning. Unless otherwise mentioned, graphs were visualized using ggplot2. Correlations of categorical values were calculated using Cramer's V and a correlation matrix was computed using the corrplot package. Circos plots were created using the circlize package. Spatial maps were created using data for spatial visualization of Luxembourg publicly available through a national data platform and the geojsonio package.

For all significance tests, the *p*‐values were set to: **** <0.0001, *** <0.001, ** <0.01, * <0.05 and ns ≥ 0.05.

## 3 RESULTS

### 3.1 A high allergy prevalence matched to allergic sensitization rates and impairment of health

**FIGURE 1:**
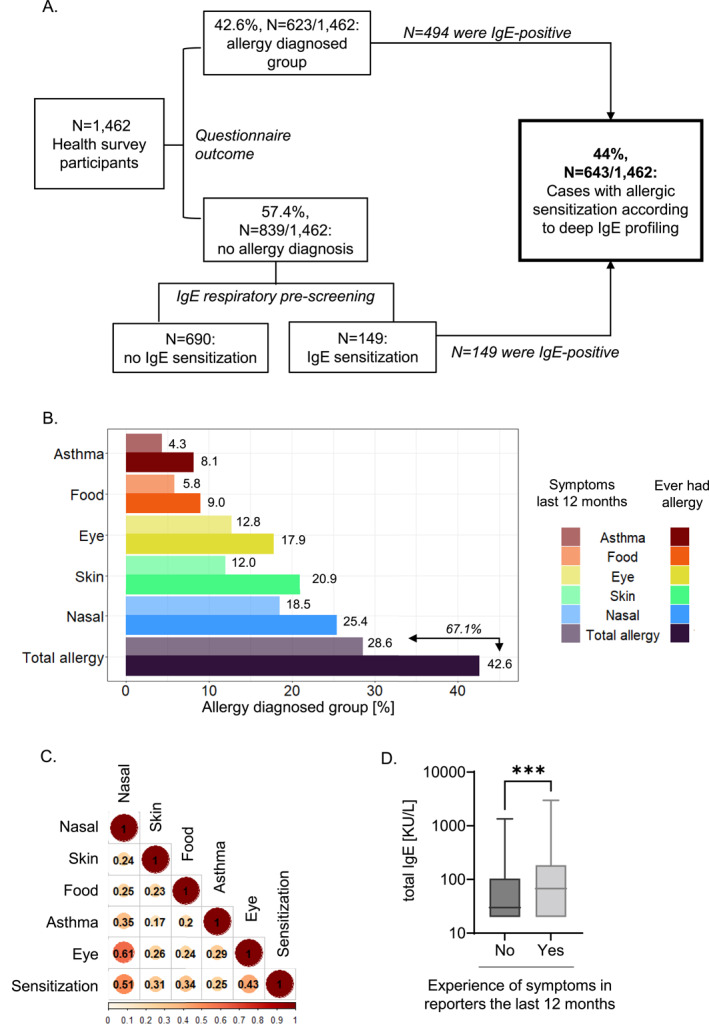
(A) Workflow of the study and overview of the results. (B) The percentage of participants (*N* = 1462) reporting physician‐diagnosed allergy (“diagnosed group”) with recent symptoms (half‐tone colors) and symptoms ever in life (bright colors). Indicated are reported allergies of any kind (purple), nasal allergy (blue), skin allergy (green), eye allergy (yellow), food allergy (orange) and asthma (red). (C) The correlation matrix comparing allergy report and sensitization (diagnosed group, *N* = 623). Correlation of categorical values was calculated using Cramer's V. Correlation coefficients between 0 and 0.19 indicate no correlation and 0.2–0.29 weak, 0.30–0.49 moderate, 0.5–0.69 strong, and 0.7–1 very strong correlation. (D) Total IgE levels of participants reporting recent allergy symptoms (light gray; *N* = 332) versus those reporting allergy symptoms a long time ago (dark gray; *N* = 153), *p*‐value calculated using Mann‐Whitney testing. For all significance tests, the *p*‐values were set to: **** <0.0001, *** <0.001, ** <0.01, * <0.05 and ns ≥ 0.05.

Our study cohort comprised an adult sample representative of the population of Luxembourg in terms of age, sex and district (*N* = 1462, Table 1). Many participants, 42.6% (*N* = 623/1462) in total, reported a clinical history of physician‐diagnosed allergy (hereafter called “diagnosed group or participants”) (Figure 1A; Table 1A).

**TABLE 1:** Demographics of the population‐based study cohort, the biosample group of the EHES LUX cohort (*N* = 1462), and differences in allergy diagnosed versus non‐diagnosed participants.

A. General demographics			
Variable	Signifier	Number of participants	Number of participants [%]
Sex	Male	691	47.3
	Female	771	52.7
Age	Mean [years] (SD, min–max)	44.9 (10.1, 26–65)	NA
	Median [years]	45	NA
	25–34	224	15.3
	35–44	412	28.2
	45–54	396	27.1
	55–65	295	20.2
Work	Capable of work	1319	90.2
	Retired/disabled	143	9.8
Country of birth	Luxembourg	761	52.1
	Neighboring countries	205	14.0
	Europe, other	379	25.9
	Non‐European	117	8.0
Have you ever had an allergy?	No	839	57.4
	Yes	623	42.6

B. Significant differences between participants reporting allergy and the rest of the cohort^a^			
Variables	Allergy diagnosis [%]	All others [%]	*p*‐value^b^
Gender			
Male	43.2	50.7	0.02932
Female	56.8	49.3	
Education level			
Primary or less	6.4	10.4	0.02575
Secondary	52.3	55.1	
Tertiary	41.1	34.0	
Absence from work due to personal health problems (last 12 months)?			
Yes	43.3	35.0	0.01836
No/I don't work	56.5	64.8	
Any time in the past 12 months when you needed health care but did not get it timely for the following reasons?			
The time needed to obtain an appointment was too long			
Yes	19.1	12.6	0.02575
No	58.1	64.2	
No need for health care	22.6	22.9	
In the past 12 months, have you had any of the following diseases or conditions?			
Severe headache such as migraine			
Yes	16.9	11.1	0.01936
No	83.1	88.9	
In the past 2 weeks, have you used any medicines that were prescribed to you by a doctor?			
Were these medications against lowering blood cholesterol level			
Yes	7.7	11.6	0.01911
No	92.0	87.8	

C. Reported allergy symptoms			
	Number of participants	[% cohort]	[% of allergy diagnosis]
Eye inflammation due to allergy	261	17.9	41.9
Nasal allergies including hay fever	372	25.4	59.4
Eczema or any kind of skin allergy	306	20.9	49.1
Food allergy	131	9.0	21.0
Asthma	119	8.1	19.1
Allergy symptoms in the past 12 months			
No	1044	71.4	NA
Yes	418	28.6	67.1
Eye inflammation due to allergy	187	12.8	30.0
Nasal allergies including hay fever	271	18.5	43.5
Eczema or any kind of skin allergy	176	12.0	28.3
Food allergy	85	5.8	13.6
Asthma	63	4.3	10.1

D. IgE sensitization			
	Number of participants	[% cohort]	[% of allergy diagnosis]
Overall sensitization^c^	643	44.0	77.8

E. Total IgE			
	All sensitized	Allergy symptoms within the last 12 months	No allergy symptoms within the last 12 months
Mean total IgE [kU/L]	138.6	168.1	105.3
Range of total IgE [kU/L]^d^	20–2979	20–2979	20–1779

Abbreviations: EHES LUX, European Health Examination Survey in Luxembourg; sIgE, specific immunoglobulin E.

^a^ Report on ever having been diagnosed with an allergy, as well as all allergy subtypes, and having an allergy or any allergy subtype in the last 12 months was highly significant with *p* < 0.0001 for all variables.

^b^ *p*‐values were calculated using Chi‐square for categorical values, Fisher's exact *t*‐test for continuous values, followed by Benjamini‐Hochberg correction; *p*‐values <0.05, significant.

^c^ Sensitization defined as significant sIgE against at least one allergen/extract of >0.3 kU_A_/L.

^d^ Twenty kU/L is the lower detection limit of the assay.

Using data from the EHES‐LUX health survey, we compared the diagnosed group with the rest of the cohort to profile socioeconomic and health‐related conditions in both groups. The diagnosed group was significantly more female and higher educated. They also complained of increased medical needs, with times to obtain an appointment with medical professionals being too long, and more absence from work due to personal health problems (Table 1B). They indicated more longstanding illness or health problems (39.6% vs. 33.8%) and higher prescribed medication intake (53.8% vs. 48.6%) as a trend. Thus, the diagnosed group appeared with a health‐impaired profile, pointing to the disease burden through allergies.

We looked into allergic symptoms to better understand clinical outcomes. Among the diagnosed group, most declared nasal allergies followed by skin and eye allergies (25.4%, 20.9% and 17.9%, respectively; Figure 1B; Table 1C). A fourth of the diagnosed group indicated to be diagnosed for food allergies and asthma with 9% and 8.1%, respectively. Many participants disclosed multiple organ involvement. A combination of eye and nasal allergy was reported most commonly (Figure 1C), namely by one third of the diagnosed group, pointing to respiratory conditions including seasonal or perennial allergies.^11^ Most participants of the diagnosed group, almost 70%, had any of the allergic symptoms within the last 12 months (Figure 1B; Table 1C), reflecting the chronicity of their allergic status.

We investigated whether allergy diagnosis would match to allergic sensitization, using a total IgE‐readout. Remarkably, we found an overall sensitization rate of 44.0% in our cohort (Figure 1A; Table 1D), which compares to the numbers reached in allergy diagnosis. We further correlated total serum IgE results with allergy diagnosis. Participants with recently diagnosed symptoms had higher total IgE as compared to the ones with symptoms longer time ago (*N* = 485, allergy last 12 months: mean 169.3 kU/L vs. ever had allergy: mean 104.4 kU/L; *p* = 0.0005; Figure 1D; Table 1E). As IgE might decrease in the absence of clinical signs,^12^ this was plausible and supported further our approach.

### 3.2 Respiratory allergies were predominant, elicited by both seasonal and perennial sources

As sensitization correlated with clinical symptom reports, especially for nose and eye allergy (Figure 1D), we assessed possible allergy triggers. We chose a deep sIgE‐profiling on 298 allergens to cover most sources of sensitization.

**FIGURE 2:**
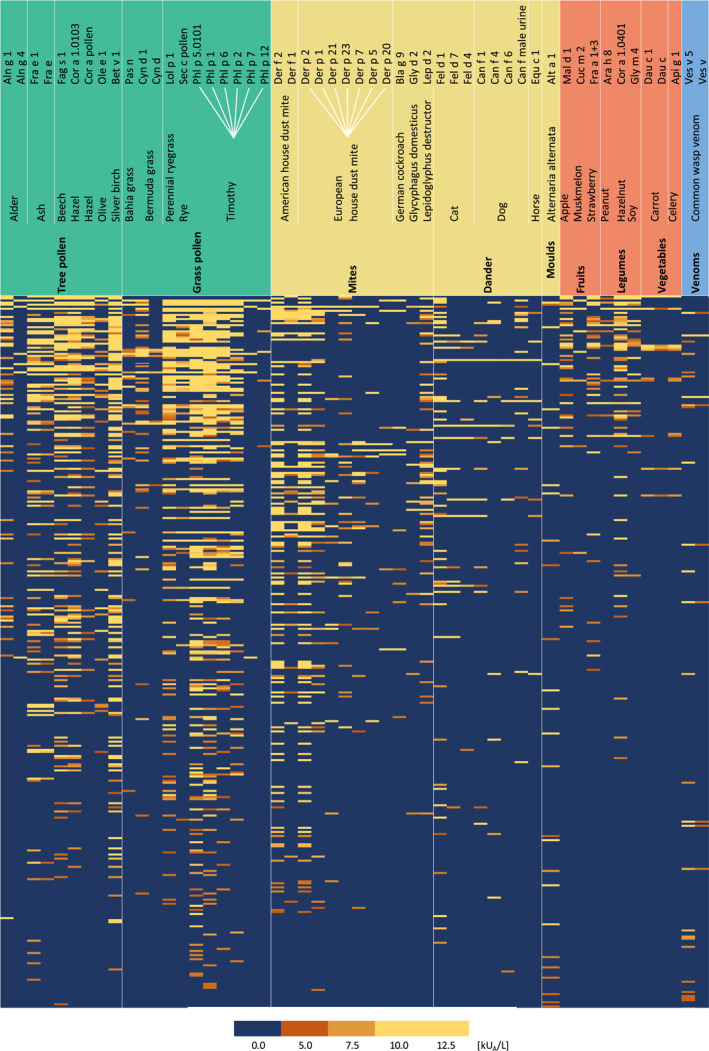
The heatmap of most eliciting sources based on specific IgE levels and population reached, selected for specific IgE >5 kU_A_/L for all participants with at least 1 allergen/extract >5 kU_A_/L. Color grading within the heatmap indicates levels of specific IgE; darkest orange color signifies 5 kU_A_/L, with the color getting lighter the higher the value is. Colored columns signify exposure route (green = outdoor exposure, yellow = indoor exposure, orange = food, blue = other exposure routes). Every column represents one allergen and every row indicates one participant, reaching this level of sensitization (*N* = 346). IgE, immunoglobulin E.

Respiratory sensitization was predominant in this representative adult cohort (Table E4). Tree pollens were the most sensitizing allergens, followed by grass pollen and mites in 52.4%, 51.8% and 40.3%, respectively, of the IgE‐positive population (Figure E2; Table E4). These allergen groups also entailed the highest IgE levels (sIgE >5 kU_A_/L: tree pollen 26.8%, grass pollen 28.0% and mites 20.7%, respectively). Allergens from food and venom sources also played a role, but to a lesser extent (Figure E2). Many participants were sensitized to more than one stimulus, both to unrelated allergen sources and to multiple allergens from the same source (Figure 2). The major birch pollen allergen Bet v 1 revealed the highest sIgE levels (mean 4.9 kU_A_/L for all sensitized and range 0–452.5 kU_A_/L) and the timothy grass allergen Phl p 1 revealed the widest spread of sensitization (61.9% sensitized in the full cohort). Co‐sensitization to 7 allergens from house dust mites and 6 allergens from timothy grass pollen emerged from the deep IgE‐typing (Figure 2; Table E5), pointing to clinical reactivities based on polyclonal immune responses.^13^, ^14^ On a molecular level, allergens belonging to the PR10 group elicited the most sensitization at elevated sensitization levels of >5 kU/L (29.9% and 19.6% of sensitized cohort, respectively). Other molecular groups causing higher levels of sensitization were allergens belonging to the NPC2 and Ole e 1 families, the 7/8S globulin family and lipocalins (15.6%, 12.1%, 7.8% and 4.5% of the sensitized population respectively). Other molecular groups, such as TLPs, profilins, albumins, nsLTPs and tropomyosins played a minor role in sensitization (Table E6).

### 3.3 Multi‐sensitization is highest in the youngest generation, urbanization as contributing factor

While the youngest participants, born in the 1980s/1990s, grew up in the middle of the first wave of the allergy epidemic, the oldest generation, born in the 1950s/1960s, experienced less lifestyle changes and biodiversity loss during adolescence.^15^ This led us to hypothesize that the youngest age group would show a different sensitization profile compared to the oldest age group.

**FIGURE 3:**
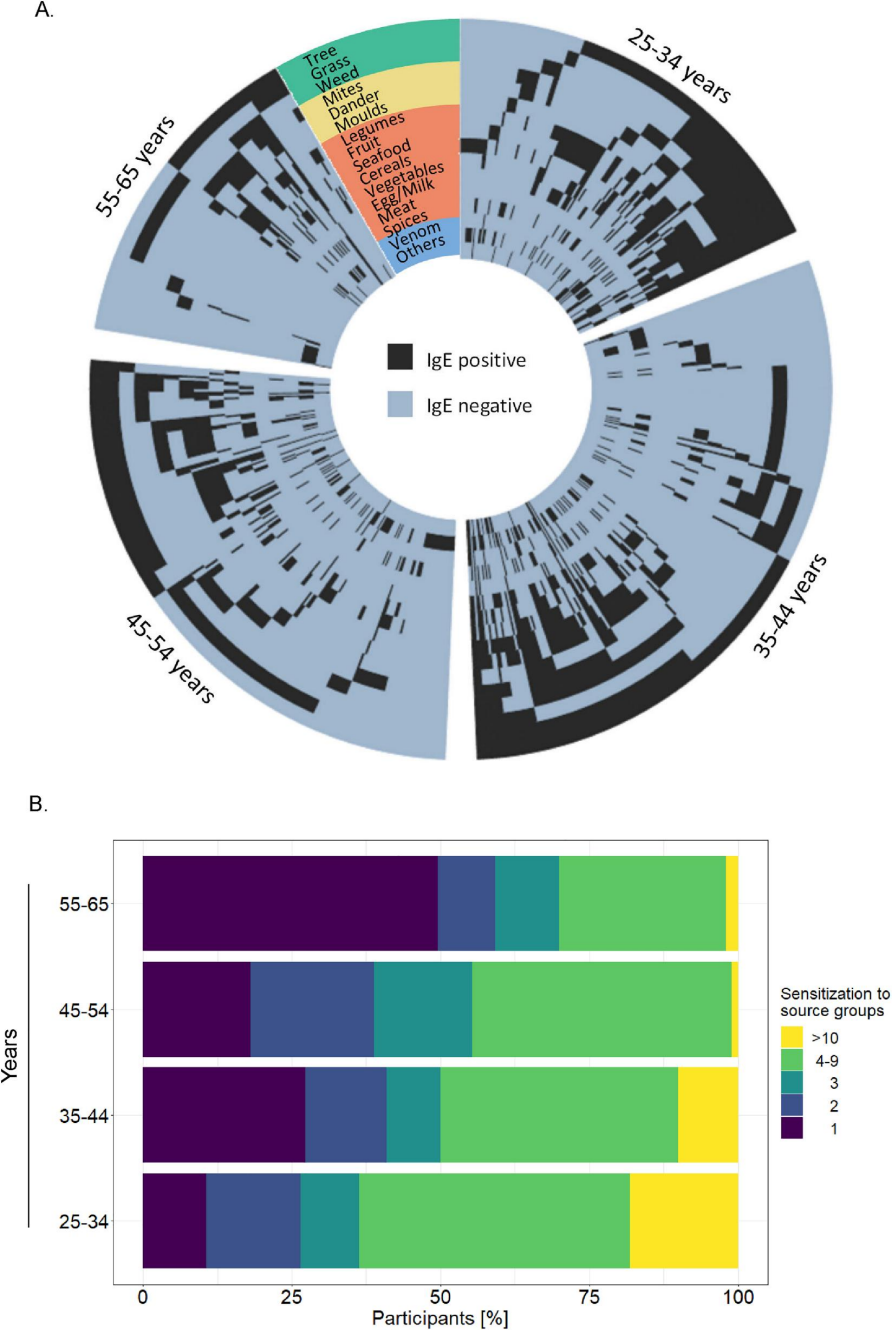
(A) Circus plot showing co‐sensitization to all allergen groups for different ages. Black boxes indicate sensitization to at least one allergen or extract in the group >0.3 kU_A_/L, and light blue boxes indicate no specific IgE against any source in that group. Every radial line indicates one participant. Colored rings signify exposure route (green = outdoor exposure, yellow = indoor exposure, orange = food, blue = other exposure routes). (B) Bar plot of co‐sensitization to multiple allergen source groups divided by age. Purple indicates mono‐sensitization, dark blue sensitization to 2 different source groups, blue to 3, green to 4–9 sources and yellow indicates strong multi‐sensitization to 10 or more sources. IgE, immunoglobulin E.

We found that the eliciting allergy sources were similar through all age groups, with tree pollen being the most sensitizing source and co‐sensitization between tree pollen and grass pollen being the most common. However, multi‐sensitization was the highest in the youngest generation (Figure 3A,B), while mono‐sensitization was most prevalent in the oldest generation (*p* < 0.0001). The age group 25–34 showed 15.9% sensitization to 2 allergen groups, 9.8% sensitization to 3, 63.6% to more than 3, while in the 55 to 65‐year‐old cohort population 9.7% were sensitized against 2 allergen groups, 10.7% against 3, 30.1% against more than 3. Differing upbringing and overall living conditions may account for the 10‐times higher poly‐sensitization in the youngest age‐group (18.2% in 25–34 vs. 1.9% in 55–65 comparing sensitization to 10 allergen groups or more, *p* = 0.0002) (Table E7). To corroborate the effects of lifestyle on sensitization patterns, we defined a group of 485 of the diagnosed group that also had sensitization to any allergen or extract <0.3 kU_A_/L, relating to 31.7% of the whole cohort. Most of the participants in this group were located in the geographical south of the country (Figure E3A,B; Table E8). While Luxembourg is a small country (stretching 82 km North–South and 57 km East–West) and therefore has little change in climate or vegetation throughout the country, the south of the country was highly industrialized due to iron ore mining.^16^, ^17^ Here, sensitization corresponds with increasing urbanization^18^ (Figure E3B; Table E8).

### 3.4 Unsupervised analysis identified distinct IgE clusters allowing advanced clinical interpretation

**FIGURE 4:**
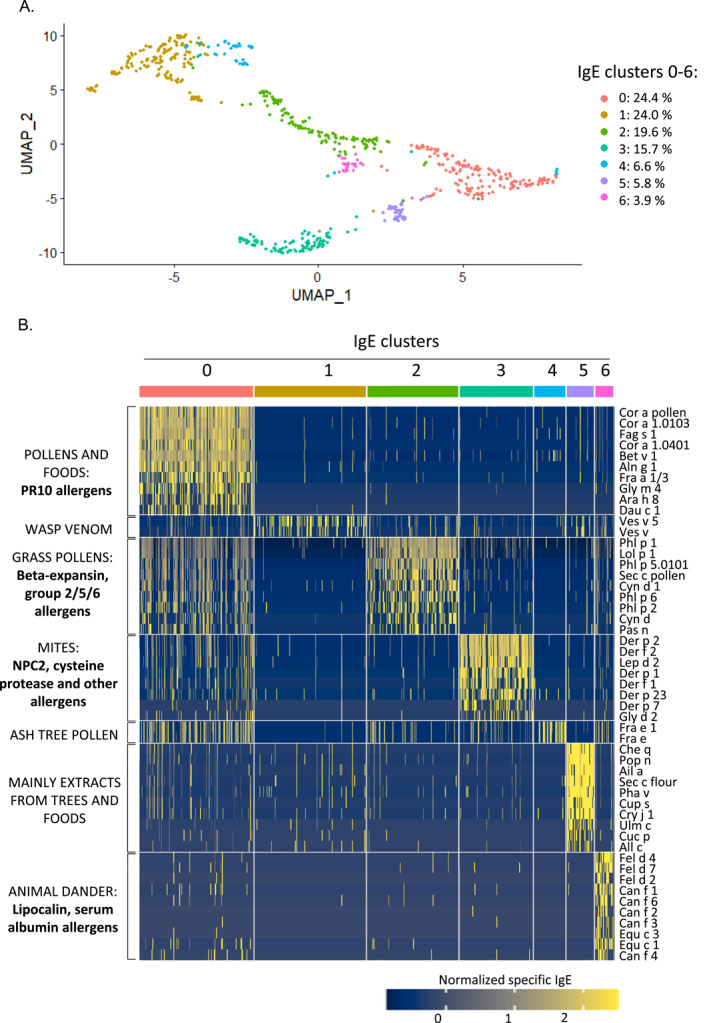
(A) Unbiased clustering of sensitized participants (*N* = 643) based on their IgE signatures over 298 allergens/extracts by UMAP. Differently colored dots indicate different clusters. Percentage represents the percent of sensitized participants clustered together. (B) IgE binding characteristics differentiating between the seven IgE clusters. Columns indicate participants, rows are sorted and selected by a maximum of the 10 top most signifying allergens. Right: indicated allergens are the ones most significantly different between the cluster and all other clusters. Left: biological origins and groups of structural homologous allergens. Color code of the heatmap indicates normalized specific IgE levels, with higher levels being brighter yellow. IgE, immunoglobulin E; NPC2, Niemann pick type C2 protein; PR‐10, pathogenesis related class‐10 like protein family; UMAP, Uniform Manifold Approximation and Projection.

We thought to take full advantage of integrating complex signatures of sIgE patterns. Our novel, unbiased clustering of sIgE‐profiles of 298 sIgE values per participant grouped participants into 7 clusters (Figure 4A).

IgE cluster 0 had two main characteristics: the highest sensitization to pathogenesis related class‐10 (PR‐10) molecules and the highest level of co‐sensitization to other main groups. Here, 24.4% of participants were gathered together. The allergens/extracts most different between this cluster and all other clusters (hereafter called “differentiating allergens”) were found for hazelnut allergens and extracts (Table E9) with an sIgE average of 11.6, 4.9 and 4.6 kUA/L for Cor a1.0103, Cor a1.0401 and the Cor a pollen extract. Amongst others, relevant PR‐10 sensitization appeared for birch (Bet v 1), beech (Fag s 1), alder (Aln g 1), soy (Gly m 4) and peanut (Ara h 8). The strawberry allergen mix Fra a 1/3 also defining this cluster consists of a mix of the PR‐10 like protein Fra a 1 and the nsLPT protein Fra a 3. That only Fra a 1 did contribute in the cluster definition would have to be further confirmed with the single molecule. Beyond those cross‐reactive marker allergens related to the pollen‐fruit‐syndrome,^19^ participants showed co‐sensitization to a great extent (Figure 4B), mainly to multiple grass pollen allergens and mite allergens. Sensitization was highest for Bet v 1, with an average of 23.2 kUA/L. Interestingly, sensitization was also high for the grass pollen allergens Phl p 1 and Phl p 5.0101 (average of 9.4 and 8.9 kUA/L respectively). Participants in this cluster had the highest total IgE values of all sensitized participants (*p* < 0.0001, average 222.8 kU/L, median 98 kU/L, 20–2274 kU/L).

IgE cluster 1 was defined by wasp venom sensitization, with the wasp venom extract Ves v and the concurring allergen Ves v 5 being the differentiating allergens. This cluster comprised 24% of sensitized participants. Here, the total IgE median was only 20 kU/L, which is the lower cut‐off of total IgE measurement. Mean sensitization against the wasp allergen was low, with an average 1.0 kU_A_/L for Ves v 5 and 0.4 kU_A_/L for Ves v extract (0–6.0 kU_A_/L for Ves v and 0–14.6 kU_A_/L for Ves v 5).

IgE cluster 2 comprised 19.6% of the sensitized study population and was defined by sensitization against grass pollen allergens and extracts. The timothy grass pollen allergens Phl p 5.0101, Phl p 1, Phl p 6 and Phl p 2 were under the most differentiating allergen features in this cluster (Table E9). Other relevant allergens were bermuda grass pollen Cyn d 1 and bermuda grass pollen extract, the rye grass pollen Lol p1, as well as others. Total IgE for participants in this cluster was in average 104.0 kU/L, with a wide range from 20 to 1346 kU/L (median 31.15 kU/L), pointing to a diverse sensitization burden within this cluster. Interestingly, sIgE levels for the marker allergen Phl p 5.0101 were in the same range as for IgE Cluster 0 (average 8.7 kU_A_/L) with lower average sIgE levels for Phl p 1, Lol p1, and Phl p 6 (average 6.6, 4.7 and 4.7 kU_A_/L, respectively).

IgE cluster 3 encompassed 15.7% of the sensitized cohort, with a clear pattern of house dust mite sensitization. European/American house dust mite allergens were the most differentiating allergens for this cluster (Der p 2, Der p 1, Der p 23, Der p 7, Der p 21, Der f 2, Der f 1), as well as the storage mite allergens Lep d 2 and Gly d 2. The average total IgE was the second highest for all clusters at 208.6 kU/L (range 20–2979 kU/L, median 53 kU/L). The main sensitizing allergens were Der p 2 and Der f 2 (average sIgE 12.2 and 12.0 kU_A_/L), with lower levels of sIgE between 4.0 and 1.1 kU_A_/L for other mite‐allergens. 76.2% of this cluster were sensitized to half or more of the signifier allergens, suggesting a higher clinical burden.^20^

IgE Clusters 4, 5 and 6 grouped 6.6%, 5.8% and 3.9% of the cohort, respectively. Briefly, IgE cluster 4 comprised participants with sensitization to ash extract and allergen Fra e and Fra e 1 that had no sensitization to most differentiating allergens. This group had the lowest average total IgE (43.9 kU/L, range 20–271 kU/L, median 20 kU/L). Cluster 5 encompasses sensitization to different tree pollen and food, *mostly derived from* extracts (total IgE average 93.6 kU/L, range 20–846 kU/L, median 20 kU/L), while participants with sensitization to animal epithelia and dander cluster together in cluster 6 (total IgE 206 kU/L, range 20–708 kU/L, median 132 kU/L). Notably, of these IgE clusters, only participants in IgE cluster 6 have a median total IgE over the cut‐off value, suggesting a higher clinical burden in this smallest group.

### 3.5 Participants' IgE signature complexity correlated with socioeconomic and clinical burden

To establish the potential health and socioeconomic burden of the above clustered participants, the health reports of participants of each IgE cluster were compared with those of the other sensitized or non‐sensitized participants on variables related to overall health and lifestyle.

**FIGURE 5:**
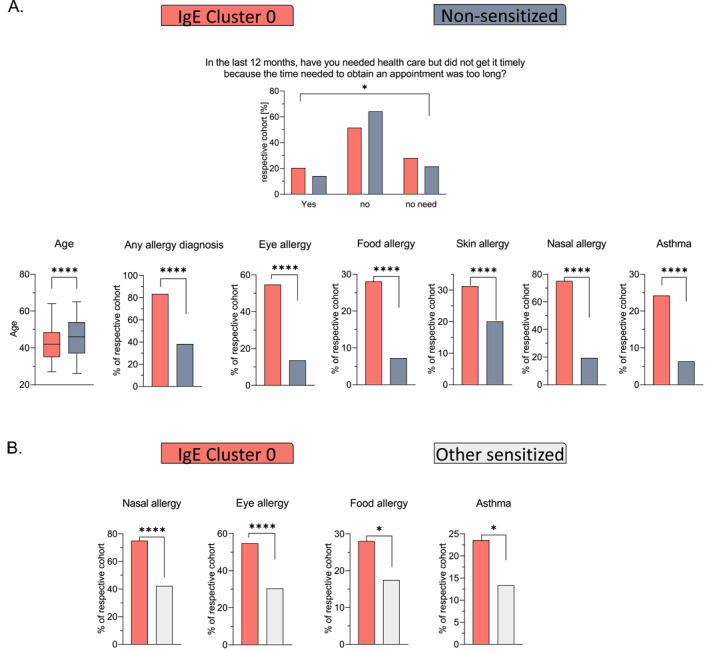
(A) Significant differences between IgE cluster 0 and the non‐sensitized population cohort on selected variables concerning health and lifestyle. Allergy diagnosis refers to all participants reporting physician‐diagnosed allergy. (B) Significant differences between IgE cluster 0 and the other sensitized cohort on variables concerning health and lifestyle. IgE, immunoglobulin E. For all significance tests, the *p*‐values were set to: **** <0.0001, *** <0.001, ** <0.01, * <0.05 and ns ≥ 0.05.

Notably, participants in the biggest cluster, IgE cluster 0, among other things reported more eye, nasal, and food allergy and asthma than others within the sensitized cohort, suggesting a high clinical burden (*p* < 0.0001, *p* < 0.0001, *p =* 0.04, *p =* 0.03 respectively, Figure 5). Compared to the non‐sensitized cohort, they reported more medical need (*P* = 0.027), and even though not significant anymore after *p*‐value correction, a higher absence from work due to health problems (43.9% vs. 37.2%) (Table E10). This clearly marks the biggest cluster of this cohort as the group not only with the highest sensitization load but accordingly with the highest need for medical attention. For the other clusters, differences between participants within a given cluster and other sensitized participants/non‐sensitized participants were less pronounced, potentially due to the low number of participants. Mostly, the results from these comparisons corroborate the clustering, for example, the participants in IgE cluster 2, defined by grass pollen sensitization reporting more eye and nasal allergy than other sensitized participants (*p =* 0.01, *p* < 0.0001 respectively). Another example would be participants in IgE cluster 3, the house dust mite cluster. Those participants were mostly males (*p =* 0.04). Indeed, an association between male sex and sensitization to D pteronyssinus and D farina had been demonstrated before.^21^

## 4 DISCUSSION

To our knowledge, this is the first study using a well‐established cross‐sectional population‐based health survey together with deep IgE‐profiling (184 molecular allergens, 116 extracts) and unbiased unsupervised non‐limiting clustering to investigate connections between sensitization patterns and medical needs.

Within the EHES‐LUX‐cohort, physician‐diagnosed allergy report was high, with 42.6% of the participants reporting having ever had any type of allergy. These numbers are comparable to other findings in middle and northern Europe.^18^, ^22^ Using a combinatory panel of allergens, Melén et al. reported in the 24 years‐old participants of the Swedish Children, Allergy, Milieu, Stockholm, Epidemiology (BAMSE) cohort a similarly high prevalence of sensitized participants (43.4%).^22^ Participants having reported a physician diagnosed allergy were mostly female and higher educated, which is in line with findings from Germany and Finland.^18^, ^23^ Participants who reported an allergy also reported unmet medical needs and more work absence due to health problems, which is a strain not only on the individual but also on society at large. Estimates on an EU level suggest a cost of 2405 Euro per patient insufficiently treated for allergic disease.^5^ Extrapolating average populations of 25–65 year olds in 2013–2015 together with the percentage of allergy reporters also reporting an unmet medical need, this would amount to a cost of 61.6 million euro per year in avoidable cost in Luxembourg, only for this age bracket. The allerrgic disease trajectory has likely increased since the end of the study, and the strain on society is still potentially higher.

Participants were overwhelmingly sensitized to respiratory allergens, both seasonal and perpetual (Figure E2; e.g. 52.4% tree pollen, 40.3% house dust mites). This fits with findings in the adult population of the BAMSE cohort, where sensitization to airborne allergens (grass/tree pollens, dander, and mites) was high.^22^ Others revealed similar findings in adolescent participants of the Manchester Asthma and Allergy Study and the Multicentre Allergy Study (MAS) birth cohort, respectively.^24^, ^25^ Indeed, several birth cohort studies did focus on analyzing IgE responses in correlation with clinical symptoms and in a time‐resolved mode to identify risk molecules and predictors of persistency of progression of respiratory diseases into adolescence and early adulthood.^22^, ^25^, ^26^, ^27^, ^28^ As a continuation of those, this study will be discussed as a follow‐up, which is our population in the age group of 26–65 years from another geographical region in Europe.

For respiratory allergens, our participants often showed sensitization to multiple molecular allergens per group suggesting high clinical reactivity, such as for grass pollen with complex IgE patterns for the allergens Phl p 1, 2, 5 and 6 (Figure 2). The evolution of antibody responses to complex patterns, known as “molecular spreading,” has been described in birth cohorts using molecule‐resolved diagnosis. For grass pollen allergy, this was linked to the initiator allergen Phl p 1 followed by expansion to Phl p 4, 5 and others.^25^, ^29^ We could confirm such patterns of molecular spreading in our cohort. For grass‐pollen sensitized participants, grouped in IgE clusters 0 and 2 (Figure 4), we detected complex late stage sensitization profiles (e.g., Phl p 1, Phl p 2, Phl p 5, Phl p 6), as, for instance, reported in the longitudinal follow‐up of the MAS cohort. Also, we could confirm molecular polysensitization for house dust mite allergens at adult age, with the characteristic IgE signature to Der p 1, Der p 2, Der p 23, and Der p 7 described previously in adolescence.^30^ Of note, all initiator allergens (e.g., Phl p 1, Bet v 1, Der p 2) scored decidedly high in our unsupervised clustering approach to determine cluster definitions, which further corroborates their central role in atopy progression.^29^

Poly‐sensitization was especially high in the youngest age group (Figure 3). This corroborates findings from Beutner et al., who showed age‐related progression to aeroallergens.^31^ Polysensitization has previously been correlated with more severe symptoms and reduced working efficiency.^32^ This increasing burden of sensitization may suggest an increasing burden on the health system, with younger generations generating more socioeconomic cost.

Our unsupervised clustering approach revealed 7 different clusters, most of which were defined by respiratory molecular allergens (Figure 4).

Indeed, respiratory allergens appear to play an important role in the trajectories of allergic diseases from child to adulthood. Wickman et al. described such risk allergens as early predictors of respiratory disease at the age of 16 years, using two birth cohorts.^26^ Those risk molecules are also key signifier allergens in the computational IgE clusters of our adult cohort (Bet v 1 in cluster 0; Phl p 1 in clusters 0 and 2; Der p 1/Der f 2 in clusters 0 and 3), corroborating such molecular patterns of persistent respiratory disease. Previous studies have used other computational approaches of component‐specific sensitization patterns, using different statistical models and allergen panels in pediatric participants.^24^, ^27^, ^33^ Based on cross‐sectional analyses in 11 years old children from a population‐based birth cohort, Fontanella et al. combined several machine learning approaches to resolve seven distinct clusters of IgE responses on the basis of 44 allergens.^27^ The connectivity structure of those IgE clusters matched with allergens’ protein homology and/or the biological source. This concurs with our findings, such as for clusters 2, 3 and 6, where decisive allergens were PR10 (e.g. Cor a 1, Bet v 1, Gly m 4) , house dust mite (e.g. Der p 1, Der p 2) and animal dander allergens (e.g., Feld d 4, Can f 1), respectively. The 11 singletons, meaning clusters only consisting of one allergen.^27^ We did not see in our study because of our different clustering approach. Interestingly, their network analyses described the high connectivity of the PR‐10 cluster to many other clusters. We could confirm this central role, as signifier PR‐10 allergens only appeared together with other allergens in cluster 0 instead of forming their own isolated cluster.

In our study, the main IgE clusters 0, 1, 2, and 3 encompass 83.7% of the sensitized population and are defined by respiratory allergens/extracts and structurally similar food allergens as well as insect venoms. For IgE cluster 1, comparable levels of venom sensitization have been found in a German cohort of a similar sized population, as well as in other European studies.^34^, ^35^, ^36^ This study also showed that overall high sensitization to venom allergens may not correlate with clinical reactivity after being stung. Conversely, low sIgE levels against wasp venom may still lead to an anaphylactic reaction to a sting, possibly due to wash out over time, making extrapolation from sensitization to clinical reaction difficult.^37^ It has previously been reported that the concentrations of allergen‐specific IgE relative to non–allergen‐specific IgE correlate with effector cell reactivity, correlating better with clinical reactivity than sIgE or total IgE levels alone.^38^, ^39^, ^40^, ^41^ Such ratios have also been suggested as a good predictor of reaction severity and treatment success in venom allergy.^42^, ^43^ Calculating IgE specific activity based on the previously established formula^41^, ^42^ “sIgE to Ves v 5 or wasp venom extract/total IgE × 100,” we found 2.85% (0.0–37.45) for Ves v 5% and 1.17% (0.0–25.30) for wasp extract. We found that 34% (52/152) had elevated and 7% (10/154) even more elevated sIgE/total IgE ratios that pointed to clinical reactivity and even systemic reactions, respectively, in comparison to previous findings.^39^, ^41^, ^42^

IgE cluster 4 was defined by sensitization to Ash tree pollen allergen and extract. Interestingly, sensitization against the main olive tree allergen Ole e 1 was not differentiating enough between clusters to be classified as a stratifying allergen, even though it belongs to the same group of Oleaceae family. This might be explained by the clustering approach considering IgE positivity as well as IgE levels and global patterns. While 28.9% of Fra e 1 sensitized participants had no Ole e 1 sensitization, Fra e 1 also elicited sIgE levels three times as high as Ole e 1. Therefore, Fra e 1 was determined as a differentiating allergen.

For IgE clusters 0, 2 and 3 the average total IgE levels of the participants of each cluster as well as the average levels of sIgE for the differentiating allergens of each cluster mark these participants to be clinically reactive.^44^ Remarkably, the biggest cluster, encompassing one fourth of all sensitized participants, was defined not only by allergens from the PR10 protein family but also by sensitization against airborne signifier allergens from other clusters (e.g.. grass pollen allergens Phl p 1, Lol p1, Phl p 5; house dust mite allergens Der p 2, Der f 2, Der p 1). Another study on unsupervised clustering described such complex molecular profiles yet to be present in childhood among other heterogeneous allergen profiles.^27^ Indeed, our cluster 0 represents a participant group with intensive molecular spreading to multiple allergen sources, a subgroup of patients with pollinosis combined with other respiratory diseases, as a consequence of a sequential broadening of the IgE response. Of note, only on the basis of molecule‐resolved IgE patterns we were able to align our findings to other studies, which showed earlier such characteristic endotypes.^29^

Poly‐sensitization to inhalant allergens from tree or grass pollen as well as house dust mite and animal dander has been reported before, with 70% of birch pollen allergic adults in the UK also having allergic reactions against foods.^45^ Fittingly, participants of our complex IgE cluster 0 also had clearly more allergy symptoms than all other participants, similarly as reported by Fontanella et al. for multi‐sensitized children with greatly elevated risk for asthma and wheezing,^27^ as well as our cluster 0 had the highest medical need among the sensitized participants. Previous studies assessed a lack of sufficient treatment for 90% of patients afflicted with airway or skin allergy.^5^ For participants of this cluster the diagnostic arrays with a wide panel of allergens might have been important to reveal the extremely wide polysensitization profile to main groups of airborne allergens but also to explore their therapeutic options.^46^ Regarding allergen immunotherapy (AIT), the prospect of success is considered limited given the fact that many different genuine allergens would have to be administered.^29^ Indeed, single‐AIT appears to be more effective than multi‐AIT, especially in patients with respiratory allergies and wide IgE repertoire spreads.^47^, ^48^ In such multimorbid conditions, the use of an anti‐IgE treatment appears preferable, with the potential to increase the efficacy of AIT.^48^ For clusters 2 and 3, participants with oligosensitization to mainly one allergenic source group, the molecule‐resolved IgE‐typing is of relevance as well because they are candidates for AIT. This might be tailored to the disease‐triggering sources, grass pollens and house dust mites, which were identified using the marker allergens Phl p 1/Phl p 5/Phl p 6 and Der p 1/Der f 2/Der p 23, respectively.

Our study has several strengths and limitations. We used a respiratory prescreening IgE test to select samples for IgE array analyses, which is an approach also used by others.^22^, ^28^ We cannot exclude that we had a bias and missed a participant subgroup (e.g. food‐allergic individuals) because of our preselection strategy. Another limitation of the IgE array is that the quantification of total IgE is only semi‐quantitative, which could be another bias for the interpretation of the clusters. Also, it might have been beneficial if the participant had been seen by a specialist to better detail the conditions of allergic disease (e.g. skin allergy). The strengths of our study relate to the deep population‐based knowledge database of our participants and the deep IgE panel used for studying complex IgE profiles. In comparison to the Immuno Solid‐phase Allergen Chip, the Alex2 macroarray offers an additional 200 data points. Those additional data points contributed to defining our IgE clusters, such as Fag s 1, Cor a 1.0103 and Fra a 1/3 for cluster 0 or Der f 2, Der p 23, Der p 7 and Gly d 2 for cluster 3. Finally, only our molecule‐resolved approach made this study comparable to others, including landmark birth cohorts,^22^, ^27^, ^28^ to address allergies on a lifetime axis.

In our cohort, high sensitization and allergy reports, together with significant medical need, show the urgency to decrease the impact of allergies on a personal and a socioeconomic level. Unsupervised clustering of large sIgE datasets allowed the identification of a group of participants with the highest burden by their sensitization patterns. Multifaceted computation‐based methods may in future lead to a more targeted clinical supervision of patients.^49^, ^50^

A recent study on 9 childhood cohorts showed sensitization in the context of regionality. IgE profiles varied depending on the geographical exposome of participants.^51^ Large data studies like this one may especially profit from unsupervised approaches to further explore connections between sensitization patterns and external factors. Combining algorithms for biological samples with novel approaches to digital health readouts may further improve the quality of life.^52^

Recent results from the Finnish allergy program show promising results in mitigating stressors leading to sensitization and also the need for country‐wide intervention.^53^ For Luxembourg, similar strategies will need to be developed to account for the mixed sensitization profiles of the population and the increasing sensitization in younger generations.

## AUTHOR CONTRIBUTIONS

Rebecca Czolk wrote the manuscript and analyzed the data. Oliver Hunewald established the data clustering approach. Naphisabet Wanniang helped with clinical interpretation. Gwenaëlle Le Coroller provided support for the statistical analysis and accessed and verified the data. Maria Ruiz‐Castell, Michel Vaillant and Guy Fagherazzi managed the EHES‐LUX cohort. Annette Kuehn developed the study concept and supervised and coordinated the study. Christiane Hilger, Françoise Morel‐Codreanu and Markus Ollert revised the study concept and manuscript. All authors provided critical feedback on the manuscript concept and scientific content.

## CONFLICT OF INTEREST STATEMENT

The authors declare that they have no relevant conflicts of interest.

## Supporting information

Supporting Information S1

## Data Availability

The data that support the findings of this study are available on request from the corresponding author. The data are not publicly available due to privacy or ethical restrictions.
