# Supplementary material for: Novel, computational IgE‐clustering in a population‐based cross‐sectional study: Mapping the allergy burden
Source: Clin Transl Allergy. 2023 Sep 5;13(9):e12292. doi: 10.1002/clt2.12292 (PMC10478827; doi:10.1002/clt2.12292)
Supplement: Supplementary file 1 — Supporting Information S1 [file CLT2-13-e12292-s001.docx]

**Submission to:** Clinical and Translational Allergy

**Manuscript type:** Original article

**Title:** Novel, computational IgE-clustering in a population-based cross-sectional study: mapping the allergy burden

**Running title:** Allergy burden in Luxembourg adults.

**Authors:** Rebecca Czolk^1,2^, Maria Ruiz-Castell^3^, Oliver Hunewald ^1^, Naphisabet Wanniang ^1,2^, Gwenaëlle Le Coroller^4^, Christiane Hilger^1^, Michel Vaillant^4^, Guy Fagherazzi^3^, Françoise Morel-Codreanu^5^, Markus Ollert^1,6#^, Annette Kuehn^1^*^#^.

#These authors contributed equally

*Corresponding author

**Affiliations:**

^1^ Department of Infection and Immunity, Luxembourg Institute of Health, Esch-sur-Alzette, Luxembourg.

^2^ Faculty of Science, Technology and Medicine, University of Luxembourg, Esch-sur-Alzette, Luxembourg.

^3^ Department of Precision Health, Epidemiology and Public Health Research Unit, Luxembourg Institute of Health, Strassen, Luxembourg.

^4^ Competence Center for Methodology and Statistics, Translational Medicine Operations Hub, Luxembourg Institute of Health, Strassen, Luxembourg

^5^ Department of Allergology and Immunology, Centre Hospitalier de Luxembourg-Kanner Klinik, Luxembourg.

^6^ Department of Dermatology and Allergy Center, Odense Research Center for Anaphylaxis, Odense University Hospital, University of Southern Denmark, Odense, Denmark.

**Corresponding author**

Annette Kuehn, PhD

Department of Infection and Immunity, Luxembourg Institute of Health

29, rue Henri Koch, L-4354 Esch-sur-Alzette

Telephone: + 352 26970 335, Fax: + 352 26970 390, E-mail: [annette.kuehn@lih.lu](mailto:annette.kuehn@lih.lu)

**Supplementary Methods**

***Study cohort***

The questionnaire of EHES-LUX, a national health survey on a representative Luxembourg cohort, covered a large portfolio of 489 health, lifestyle and environment determinants, as described before^1^. This study was approved by the National Ethics Committee of Luxembourg (Comité National d’Ethique de Recherche – CNER), CNER Approval N° 201205/07. All study subjects gave written informed consent for their participation in the study. Here, 165 variables relevant to health aspects were selected (Table E2), excluding variables targeting subgroups (e.g. women’s health or hard drug use). Briefly, those variables related to the participant’s social and environmental (work, private) parameters, their education, general health/chronic diseases as well as healthcare determinants. All 165 variables where used for statistical analysis. Sera samples available from 1,462 participants were applied for IgE testing (Figure E1). Statistical analysis of all 165 variables ensured that no bias was introduced by biosample availability, calculated using Chi-square for categorical values, Fisher’s exact t-test for continuous values, followed by Benjamini-Hochberg correction. Allergy report was established based on the questionnaire questions on ever or within the last 12 months having had any kind of physician-diagnosed allergy, or one of the allergy types in the report. Hereafter, participants who reported a physician-diagnosed allergy are called “diagnosed group/participants”. These reported allergy types were defined by the questions as follows:

- Eye allergy: questionnaire report of “Eye inflammation due to allergy”
- Nasal allergy: questionnaire report of “Nasal allergies including hay fever”
- Skin allergy: questionnaire report of “Eczema or any kind of skin allergy”
- Food allergy: questionnaire report of “Food allergy”
- Asthma: questionnaire report of “Asthma”

***IgE screening and IgE multiplex profiling***

All participants who did not report any physician-diagnosed allergy were screened using the ImmunoCAP Phadiatop SX1 (Phadia Thermo Fisher Scientific, Uppsala, Sweden; cut-off for positivity: 0.35 kU_A_/L; performed by Laboratoires réunis, Luxembourg S.A). We did use this sIgE-screening test for 8 respiratory allergen sources (cat and dog epithelia, *Dermatophagoides pteronyssinus*, *Cladosporium* spp, Rye, Mugwort pollen, Timothy grass pollen and Birch pollen; Phadia Thermo Fisher Scientific, Uppsala, Sweden) ^2^ in order to possibly revise incorrect informations provided by the study participants or possibly detect non-symptomatic sensitization. Sera from EHES-LUX participants, who either reported physician-diagnosed allergies (diagnosed group) or were positive in the respiratory IgE screening were analyzed with the MADx “Alex2” macroarray (MacroArrayDX, Wien, Austria) according to the manufacturer’s instructions^3-5^. Briefly, 100 ul of participant serum were used for macroarray analysis. The array comprises of a panel of 116 extracts and 184 allergen molecules. Data acquisition of IgE reactivities to 300 allergen(s)/extracts was done using a charge-coupled device camera and the ImageXplorer, followed by data mining using the Raptor software. The cut-off for positivity was 0.3 kU_A_/L. As to the dynamic range, total IgE (tIgE) were semi-/quantified at ≤2 0-2,500 kU/L and sIgE at 0.3-35 kU_A_/L. Even though tIgE measurements with the Alex2 assay are semi-quantitative, previous publications found a good correlation between the tIgE measurements on the Alex2 system and the quantitative ImmunoCAP^6^. The SX1 Phadiatop assay and the Alex2 assay are not directly interchangeable in their diagnostic accuracy due to multiple reasons (e.g. extracts used, extract mixtures, detection technology), however, previous reports on good alignment between extract measures of the ImmunoCAP and Alex2^7^. Samples with sIgE levels higher 35 kU_A_/L were diluted and the assay was repeated. Five samples tested positive for the cross-reactive carbohydrate control (Hom s LF, human lactoferrin) and were removed, because sIgE levels of these participants may not be quantifiable. SIgE values for Mes a 1, the hamster allergen, were removed due to inconsistencies in the readout, leading to a total 298 allergens/extracts used for further analysis. The nomenclature of allergens and extracts was adopted from the macroarray manufacturer.

For identifying the elicitors of allergic sensitization (Figure E2, Figure 2, Figure 3), allergens and extracts were grouped based on their sources (Table E1). These sources contained molecules and extracts from the same natural origin (e.g. the venom group containing wasp venom extract Ves v and wasp venom molecules Ves v 5). However, the groups shown in Figure 4 were generated based on most differentiating allergens and extracts identified by unbiased clustering, therefore some groups are mixed molecules and extracts (eg, Cor a pollen extract and PR10 molecules clustering together), while others may contain only molecules (eg, house dust mite-related allergens, Der p 2 to Gly d 2) or mostly extracts.

**Statistical analyses and bioinformatics tools**

Computational analysis was performed in R (version 4.1.2), using R studio (version 2022.02.03+492). Tidyverse was used for data structuring and cleaning ^8^. Unless otherwise mentioned, graphs were visualized using ggplot2 (Figure 1, B.; Figure E2, A.; Figure 2; Figure 3, B.) ^9^.

The EHES-LUX survey outcome was compared for the full cohort vs the sub-cohort with available sera (N=1,529 vs N=1,462), in to ensure no bias was introduced when focusing on national sensitization prevalence based on the sub-cohort, referred to the “biosample group”. This statistical comparison was done over 165 main variables using the base R functions (Chi-square for categorical values, Fisher’s exact t-test for continuous values, followed by Benjamini-Hochberg correction). The biosample group was found to be equivalent to the whole cohort, without any differences in the main variables of the survey (data not shown). For instance, the proportion of the allergy diagnosed participants was the same for the complete study cohort (42.9%; N=656/1,529) vs the EHES-LUX biosample group (42.6%; N=623/1,462). Minor differences were interpreted as irrelevant for the present study (N=1,529 vs N=1,462: more need for dietician and higher lifetime anxiety, *P*=0.007 and *P*=0.005, respectively).

For all sensitization data, values below the cut-off of 0.3 were set to 0. Participants with at least 1 sIgE value >0.3 kU_A_/L were considered sensitized.

The correlation matrix for categorical values of diagnosed sub-types of allergies, nose, eye, skin, food, asthma, vs the allergic sensitization was calculated using Cramer’s V in base R and visualized in R using the corrplot package^10^.

The co-allergy sensitization pattern based on most common allergy elicitors (Figure 2) was created by filtering sensitization data for all allergens eliciting sIgE levels > 5 kU_A_/L in at least 10% of sensitized participants. Each rows indicates one participant.

The circus plot (Figure 3, A.) was created using the circlize package^11^. Background colors signify exposure route (green = outdoor exposure, yellow = indoor exposure, orange = food, blue = other exposure routes), Black boxes indicate sensitization to at least one allergen or extract in the group > 0.3 kU_A_/L, grey boxes indicate no sIgE against any source in that group. Every radial line indicates one participant.

Uniform Manifold Approximation and Projection (UMAP) and cluster heatmap (Figure 4) of specific IgE profiles for all sensitized participants were analyzed and visualized with the Seurat package^12^. Cluster stability was tested in 10 runs with different seeds, resulting in identical cluster sizes and signifier allergens for each run, with only the order of signifier allergens changing from run to run, making the clustering highly stable. Different cluster resolutions were tested with a resolution of 0.5 giving the clearest separation of clusters.

In Figure E3, statistical differences in the questionnaire data from cluster 0 were calculated using base R functions (Chi-square for categorical values, Fisher’s exact t-test for continuous values, followed by Benjamini-Hochberg correction) and were visualized with GraphPad Prism 9 (Graphpad Software Inc, San Diego, USA). In the age boxplot, medians are represented, boxes cover the 25^th^ to75^th^ quartile and whiskers are covering 10^th^ to 90^th^ percentile.

Spatial maps for Figure E3 were created using data for spatial visualization of Luxembourg publicly available through a national data platform and the geojsonio-Package^13,14^. Darker color of areas in Figure E3, A. indicates higher percentage of participants from that Canton being sensitized and belonging to the allergy diagnosed group. In Figure E3, B. the scale progresses from blues over greens to yellows and reds. Dark blue color indicate lowest urbanization and deep red tones indicate highest urbanization.

For all significance tests, the P-values were set to: **** <0.0001, *** <0.001, ** <0.01, * <0.05 and ns ≥ 0.05.

**literature**

1. Bocquet V, Barré J, Couffignal S, et al. Study design and characteristics of the Luxembourg European Health Examination Survey (EHES-LUX). *BMC Public Health.* 2018;18(1):1169.

2. Zeng G, Hu H, Zheng P, et al. The practical benefit of Phadiatop test as the first-line in vitro allergen-specific immunoglobulin E (sIgE) screening of aeroallergens among Chinese asthmatics: a validation study. *Ann Transl Med.* 2018;6(8):151-151.

3. Klueber J, Czolk R, Codreanu‐Morel F, et al. High‐dimensional immune profiles correlate with phenotypes of peanut allergy during food‐allergic reactions. *Allergy.* 2022;78(4):1020-1035.

4. Feuerecker M, Strewe C, Aumayr M, et al. One Year in the Extreme Isolation of Antarctica&mdash;Is This Enough to Modulate an &ldquo;Allergic&rdquo; Sensitization? *Biomedicines.* 2022;10(2):448.

5. Platteel ACM, van der Pol P, Murk J-L, et al. A comprehensive comparison between ISAC and ALEX<sup>2</sup> multiplex test systems. *Clinical Chemistry and Laboratory Medicine (CCLM).* 2022;60(7):1046-1052.

6. Quan PL, Sabaté-Brescó M, D'Amelio CM, et al. Validation of a commercial allergen microarray platform for specific immunoglobulin E detection of respiratory and plant food allergens. *Ann Allergy Asthma Immunol.* 2022;128(3):283-290.e284.

7. Hoang JA, Celik A, Lupinek C, et al. Modeling the conversion between specific IgE test platforms for nut allergens in children and adolescents. *Allergy.* 2021;76(3):831-841.

8. Wickham H, Averick M, Bryan J, et al. Welcome to the Tidyverse. *Journal of Open Source Software.* 2019;4(43):1686.

9. Wickham H. ggplot2. In: Springer New York; 2009.

10. *R package "corrplot": Visualization of a Correlation Matrix* [computer program]. Version (Version 0.84). 2017.

11. Gu Z, Gu L, Eils R, Schlesner M, Brors B. <i>circlize</i> implements and enhances circular visualization in R. *Bioinformatics.* 2014;30(19):2811-2812.

12. Hao Y, Hao S, Andersen-Nissen E, et al. Integrated analysis of multimodal single-cell data. *Cell.* 2021;184(13):3573-3587.e3529.

13. *geojsonio: Convert Data from and to 'GeoJSON' or 'TopoJSON'* [computer program].

14. Luxembourg LadG-Dd. 2022; https://data.public.lu/en/datasets/limites-administratives-du-grand-duche-de-luxembourg.

**Online Repository FIGURES**

**Figure E1.** Technical workflow of the study. Allergy diagnosis refers to all participants reporting physician-diagnosed allergy.

**Figure E2.** Percentage of participants sensitized by allergen group. Background colors signify exposure route (green = outdoor exposure, yellow = indoor exposure, orange = food, blue = other exposure routes), colors in the bars indicate level of kU_A_/L. The grey tones of the stacked columns reflect specific IgE titers for at least one allergen/extract of the indicated group.

**Figure E3.** Spatial distribution of **A.** Participants who reported allergy and were found to be sensitized to any allergen (>0.3 kU_A_/L). Darker color indicates higher percentage of that canton being sensitized & diagnosed with allergy. **B.** Illustrates urbanization in Luxembourg, defined by the average population per canton within the study years 2013-2015. The scale progresses from blues over greens to yellows and reds. Dark blue color indicate lowest urbanization and deep red tones indicate highest urbanization. Numbers indicate the cantons, with 1: Esch-sur-Alzette, 2: Remich, 3: Capellen, 4: Luxembourg, 5: Grevenmacher, 6: Redange, 7: Mersch, 8: Echternach, 9: Diekirch, 10: Wiltz, 11: Vianden and 12: Clervaux.

**
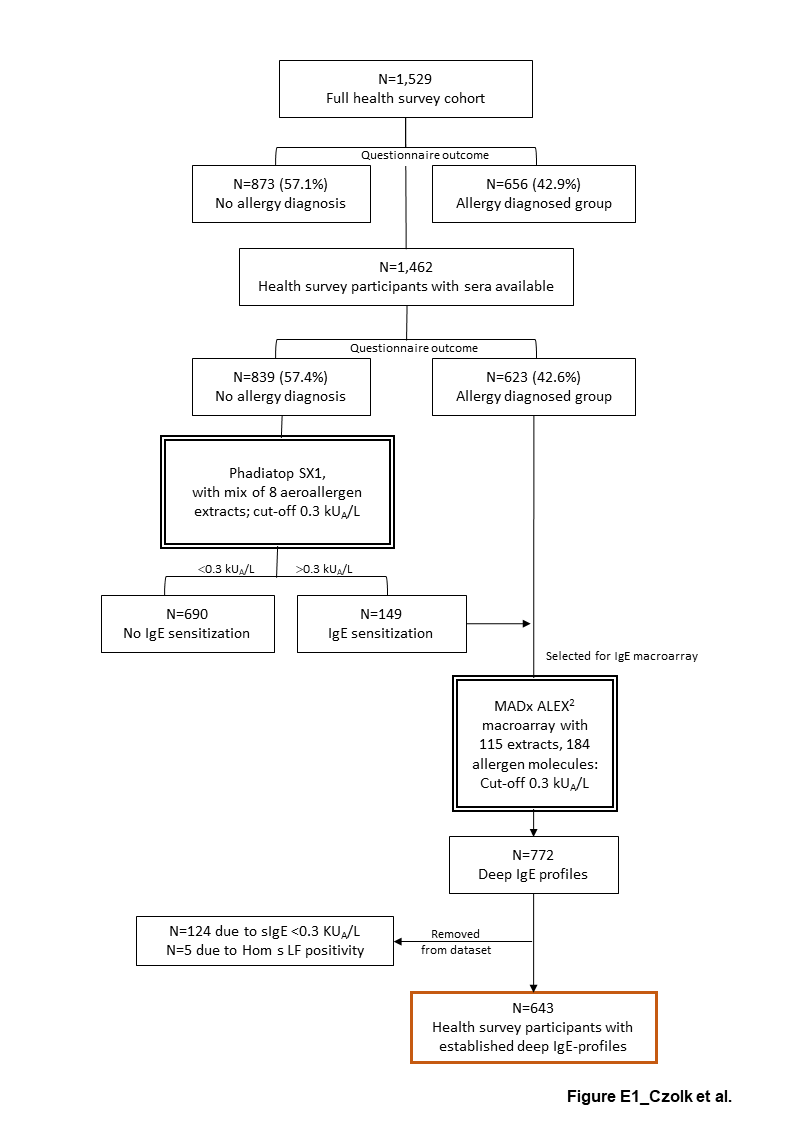

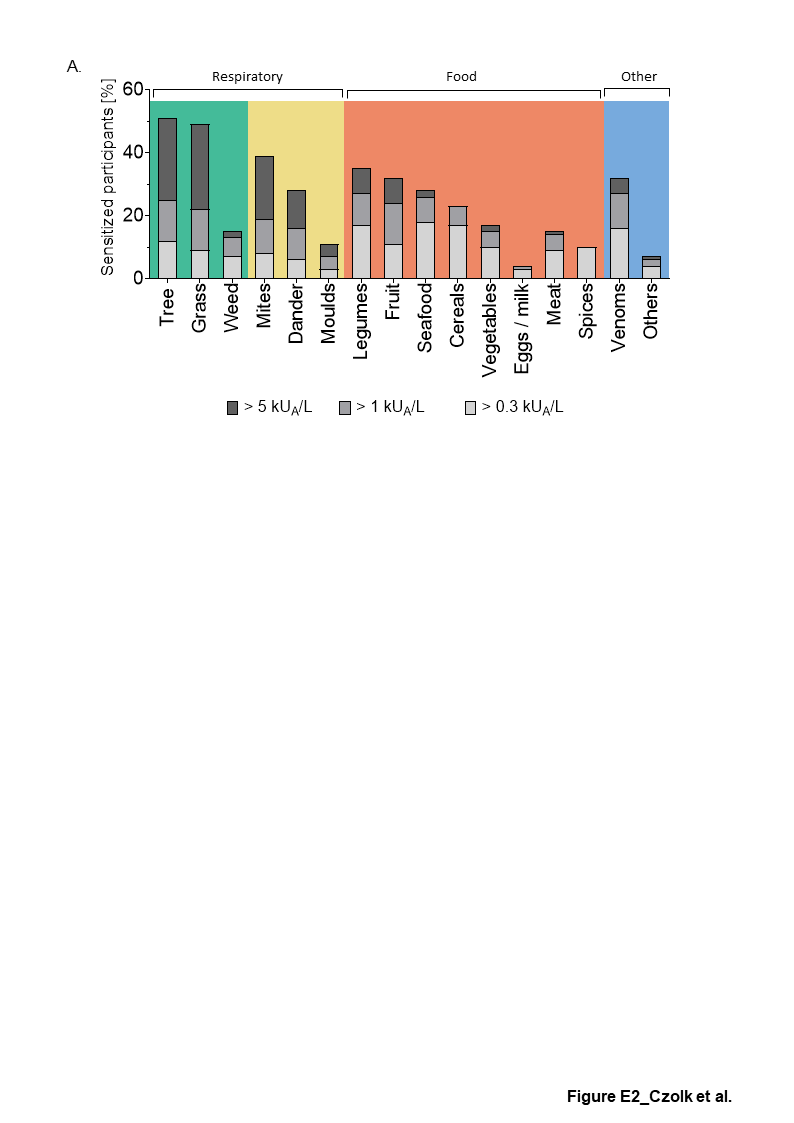

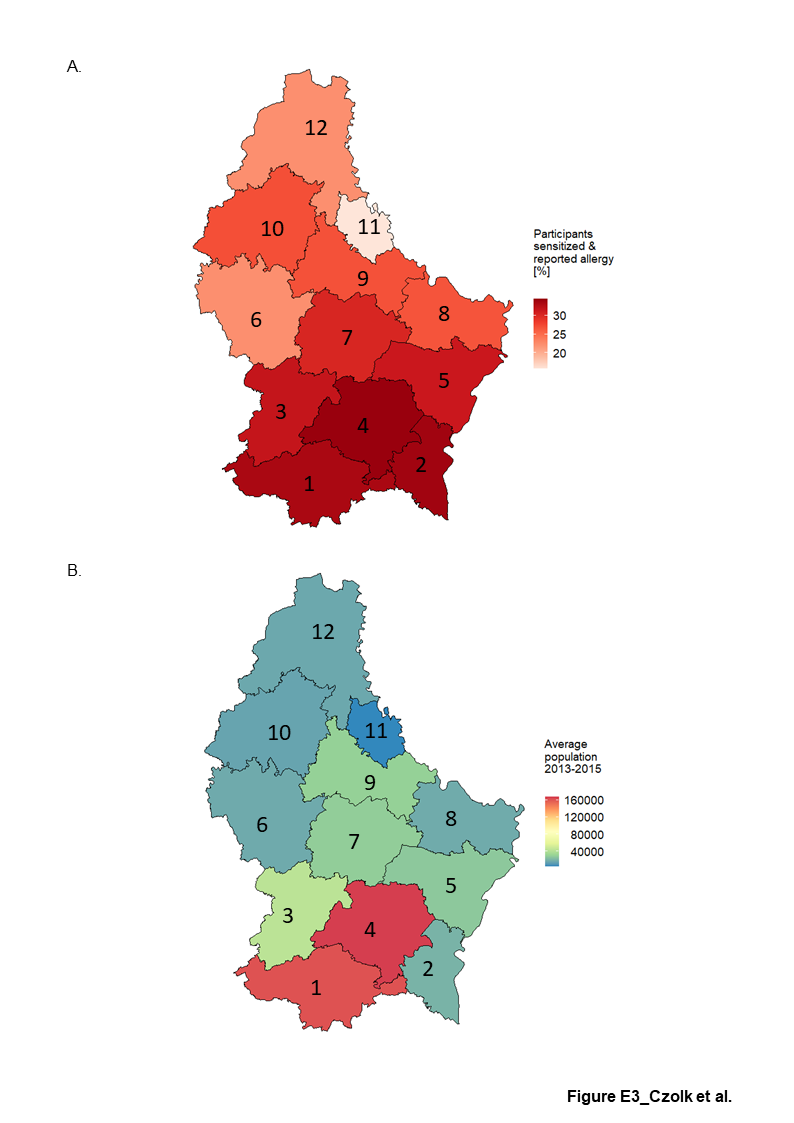
**

**Online Repository Tables**

**Table E1:** Allergen/extract group names used in the manuscript and the sources contained in these groups.

| **GROUP NAME IN MANUSCRIPT** | **SOURCES CONTAINED (Allergen/Extract identifier)** |
| --- | --- |
| Tree Pollen | *Containing allergens and extracts from tree pollen*  Acacia (Aca m), Tree of heaven (Ail a), Alder (Aln g), Silver birch (Bet v), Paper mulberry (Bro pa), Hazel (Cor a), Cedar (Cry j), Arizona cypress (Cup a), Cypress (Cup s), Beech (Fag s), Ash (Fra e), Walnut (Jug r), Mountain cedar (Jun a), Mulberry tree (Mor r), Olive (Ole e), Date palm (Pho d), London plane tree (Pla a), Cottonwood (Pop n), Elm (Ulm c) |
| Grass Pollen | *Containing allergens and extracts from grass pollen*  Bermuda grass (Cyn d), Perennial ryegrass (Lol p), Bahia grass (Pas n), Timothy (Phl p), Common reed (Phr c), Rye (Sec c) |
| Weed Pollen | *Containing allergens and extracts from weed pollen*  Pigweed (Ama r), Ragweed (Amb a), Mugwort (Art v), Hemp (Can s), Lamb’s quarter (Che a), Annual mercury (Mer a), Wall pellitory (Par j), Ribwort (Pla l), Russian Thistle (Sal k), Nettle (Urt d) |
| Mites | *Containing allergens and extracts from mites as well as cockroaches*  *Acarus siro* (Aca s), German cockroach (Bla g), *Blomia tropicalis* (Blo t), American house dust mite (Der f), European house dust mite (Der p), *Glycyphagus domesticus* (Gly d), *Lepidoglyphus destructor* (Lep d), American cockroach (Per a), *Tyrophagus putrescentiae* (Tyr p) |
| Dander | *Containing allergens and extracts from dander as well as epithelia*  Cattle (Bos d), Dog (Can f), Goat (Cap h), Guinea pig (Cav p), Horse (Equ c), Cat (Fel d), Mouse (Mus m), Rabbit (Ory c), Sheep (Ovi a), Djungarian hamster (Phod s), Rat (Rat n), Pig (Sus d) |
| Moulds | *Containing allergens and extracts from moulds as well as yeasts*  *Alternaria alternate* (Alt a), *Aspergillus fumigatus* (Asp f), *Cladosporium herbarum* (Cla h), *Malassezia sympodialis* (Mala s), *Penicilium chrysogenum* (Pen ch) |
| Legumes | *Containing allergens and extracts from legumes as well as nuts*  Cashew (Ana o), Peanut (Ara h), Brazil nut (Ber e), Pecan (Car i), Chickpea (Cic a), Hazelnut (Cor a), Soy (Gly m), Walnut (Jug r), Lentil (Len c), Lupine seed (Lup a), Macadamia (Mac i), Green bean (Pha v), Pea (Pis s), Pistachio (Pis v), Almond (Pru du) |
| Fruits | *Containing allergens and extracts from fruits*  Kiwi (Act d), Papaya (Car p), Orange (Cit s), Muskmelon (Cuc m), Fig (Fic b), Strawberry (Fra a), Apple (Mal d), Mango (Man i), Banana (Mus a), Avocado (Pers a), Cherry (Pru av), Peach (Pru p), Pear (Pyr c), Blueberry (Vac m), Grape (Vit v) |
| Seafood | *Containing allergens and extracts from seafood*  *Anisakis simplex* (Ani s), Crab (Chi spp), Atlantic herring (Clu h), Brown shrimp (Cra c), Carp (Cyp p), Atlantic cod (Gad m), Lobster (Hom g), Shrimp mix (Lit s), Squid (Lol spp), Common mussel (Myt e), Oyster (Ost e), Northern prawn (Pan b), Scallop (Pec spp), Black-Tiger shrimp (Pen m), Thornback ray (Raj c), Venus clam (Rud spp), Salmon (Sal s), Atlantic mackerel (Sco s), Tuna (Thu a), Swordfish (Xip g) |
| Cereals | *Containing allergens and extracts from cereals as well as seeds*  Oat (Ave s), Quinoa (Che q), Pumpkin seed (Cuc p), Buckwheat (Fag e), Sunflower seed (Hel a), Barley (Hor v), Rice (Ory s), Millet (Pan m), Poppy seed (Pap s), Cultivated rye (Sec c), Sesame (Ses i), Wheat (Tri a), Fenugreek seeds (Tri fo), Spelt (Tri s), Corn (Zea m) |
| Vegetables | *Containing allergens and extracts from vegetables*  Onion (All c), Garlic (All s), Celery (Api g), Carrot (Dau c), Potato (Sol t), Tomato (Sola l) |
| Eggs / milk | *Containing allergens and extracts from eggs and milk*  Cow's milk (Bos d), Camel's milk (Cam d), Goat's milk (Cap h), Mare's milk (Equ c), Sheep's milk (Ovi a), Egg white (Gal d), Egg yolk (Gal d) |
| Meat | *Containing allergens and extracts from meat*  House cricket (Ach d), Beef (Bos d), Horse (Equ c), Chicken (Gal d), Migratory locust (Loc m), Turkey (Mel g), Rabbit (Ory c), Lamb (Ovi a), Pig (Sus d), Mealworm (Ten m) |
| Spices | *Containing allergens and extracts spices*  Paprika (Cap a), Caraway (Car c), Oregano (Ori v), Parsley (Pet c), Anise (Pim a), Mustard (Sin a) |
| Venoms | *Containing allergens and extracts from insect venoms*  Honey bee venom (Api m), Long-headed wasp venom (Dol spp), Paper wasp venom (Pol d), Fire Ant (Sol spp), Common wasp venom (Ves v) |
| Others | *Containing allergens and extracts from other sources*  Pigeon tick (Arg r), Weeping fig (Fic c), Latex (Hev b), Baker's yeast (Sac c) |

**Table E2:** Demographics of the population-based study cohort, the full EHES LUX cohort (N=1,529), and differences in diagnosed group vs non-diagnosed group.

| **A. GENERAL DEMOGRAPHICS** | | | | | | | | | | | | | |
| --- | --- | --- | --- | --- | --- | --- | --- | --- | --- | --- | --- | --- | --- |
| **Variable** | **Signifier** | | **Number of participants** | | | | | | **[%]** | | | | |
| Sex | Male | | 722 | | | | | | 47.2 | | | | |
|  | Female | | 807 | | | | | | 52.8 | | | | |
| Age | Mean [years] (sd, min – max) | | 44.87 (10.1, 26-65) | | | | | | NA | | | | |
|  | Median [years] | | 45 | | | | | | NA | | | | |
|  | 25-34 | | 288 | | | | | | 18.8 | | | | |
|  | 35-44 | | 475 | | | | | | 31.1 | | | | |
|  | 45-54 | | 458 | | | | | | 30.0 | | | | |
|  | 55-65 | | 308 | | | | | | 20.1 | | | | |
| Work | Capable of work | | 1383 | | | | | | 90.5 | | | | |
|  | Retired / disabled | | 146 | | | | | | 9.5 | | | | |
| Country of birth | Luxembourg | | 800 | | | | | | 52.3 | | | | |
|  | Neighboring countries | | 215 | | | | | | 14.1 | | | | |
|  | Europe, other | | 390 | | | | | | 25.5 | | | | |
|  | Non-European | | 124 | | | | | | 8.1 | | | | |
| Have you ever had an allergy? | No | | 871 | | | | | | 57.0 | | | | |
|  | Yes | | 656 | | | | | | 42.9 | | | | |
| **B. SIGNIFICANT DIFFERENCES BETWEEN PARTICIPANTS REPORTING A PHYSICIAN DIAGNOSED ALLERGY AND THE REST OF THE COHORT°** | | | | | | | | | | | | | |
| **Variables** | | | | **Allergy diagnosed [%]** | | | | **All others [%]** | | | | | **P value*** |
| Gender | | Male | | 42.5 | | | | 50.6 | | | | | 0.02019 |
|  |  | Female | | 57.5 | | | | 49.4 | | | | |  |
| Education level | | Primary or less | | 6.1 | | | | 10.1 | | | | | 0.02827 |
|  |  | Secondary | | 53.2 | | | | 55.2 | | | | |  |
|  |  | Tertiary | | 40.5 | | | | 34.2 | | | | |  |
| Absence from work due to personal health problems (last 12 months)? | | Yes | | 43.8 | | | | 35.5 | | | | | 0.01408 |
|  |  | No/I don't work | | 56.1 | | | | 64.5 | | | | |  |
| Any time in the past 12 months when you needed health care but did not get it timely for the following reasons? | | | | | | | | | | | | | |
| The time needed to obtain an appointment was too long | | Yes | | 19.5 | | | 13.0 | | | | | 0.02019 | |
|  |  | No | | 58.1 | | | 64.3 | | | | |  |  |
|  |  | No need for health care | | 22.3 | | | 22.6 | | | | |  |  |
| In the past 12 months, have you had any of the following diseases or conditions? | | | | | | | | | | | | | |
| Severe headache such as migraine | | Yes | | 20.7 | | 15.7 | | | | | 0.02019 | | |
|  |  | No | | 79.1 | | 84.3 | | | | |  |  |  |
| In the past 2 weeks, have you used any medicines that were prescribed to you by a doctor? | | | | | | | | | | | | | |
| Were these medications against lowering blood cholesterol level | | Yes | | 11.3 | | 15.0 | | | | | 0.02072 | | |
|  |  | No | | 42.2 | | 33.3 | | | | |  |  |  |
| **C. REPORTED ALLERGY SYMPTOMS** | | | | | | | | | | | | | |
|  | | **Number of participants** | | | **[% cohort]** | | | | | **[% of allergy diagnosed]** | | | |
| Eye inflammation due to allergy | | 273 | | | 17.9 | | | | | 41.6 | | | |
| Nasal allergies including hay fever | | 385 | | | 25.2 | | | | | 58.7 | | | |
| Eczema or any kind of skin allergy | | 324 | | | 21.2 | | | | | 49.4 | | | |
| Food allergy | | 143 | | | 9.4 | | | | | 21.8 | | | |
| Asthma | | 125 | | | 8.2 | | | | | 19.1 | | | |
| Allergy symptoms in the past 12 months | | | | | | | | | | | | | |
|  | | **Number of participants** | | | **[% cohort]** | | | | | **[% of allergy diagnosed]** | | | |
| No | | 1087 | | | 71.1 | | | | | NA | | | |
| Yes | | 440 | | | 28.8 | | | | | 67.0 | | | |
| Eye inflammation due to allergy | | 197 | | | 12.9 | | | | | 30.0 | | | |
| Nasal allergies including hay fever | | 283 | | | 18.5 | | | | | 43.1 | | | |
| Eczema or any kind of skin allergy | | 187 | | | 12.2 | | | | | 28.5 | | | |
| Food allergy | | 94 | | | 6.1 | | | | | 14.3 | | | |
| Asthma | | 65 | | | 4.3 | | | | | 9.9 | | | |

°Report on ever having been diagnosed with an allergy, as well as all allergy subtypes, and having an allergy or any allergy subtype in the last 12 months was highly significant with P < 0.0001 for all variables. *P-values were calculated using Chi-square for categorical values, Fisher’s exact t-test for continuous values, followed by Benjamini-Hochberg correction; P-values <0.05, significant

**Table E3.** Variables^#^ (N=165) selected from EHES-LUX codebook, used for the present study.

| SOCIAL | | | | | | | | | | | | | | | | | | | | | | | | | | | | | | | | | | | | | | | | | | | | | | | | | | |
| --- | --- | --- | --- | --- | --- | --- | --- | --- | --- | --- | --- | --- | --- | --- | --- | --- | --- | --- | --- | --- | --- | --- | --- | --- | --- | --- | --- | --- | --- | --- | --- | --- | --- | --- | --- | --- | --- | --- | --- | --- | --- | --- | --- | --- | --- | --- | --- | --- | --- | --- |
| **Gender** | | | | | | | | | | | | | Male | | | | | | | | | | | | | | | **Marital status** | | | | | | | | | | | | Not married or PAC | | | | | | | | | | |
|  |  |  |  |  |  |  |  |  |  |  |  |  | Female | | | | | | | | | | | | | | |  |  |  |  |  |  |  |  |  |  |  |  | Married or PAC | | | | | | | | | | |
| **Age** | | | | | | | | | | | | | 25-34 | | | | | | | | | | | | | | | **Country of Birth** | | | | | | | | | | | | Luxembourg | | | | | | | | | | |
|  |  |  |  |  |  |  |  |  |  |  |  |  | 35-44 | | | | | | | | | | | | | | |  |  |  |  |  |  |  |  |  |  |  |  | Neighbouring countries | | | | | | | | | | |
|  |  |  |  |  |  |  |  |  |  |  |  |  | 45-54 | | | | | | | | | | | | | | |  |  |  |  |  |  |  |  |  |  |  |  | Other countries in Europe | | | | | | | | | | |
|  |  |  |  |  |  |  |  |  |  |  |  |  | 55-64 | | | | | | | | | | | | | | |  |  |  |  |  |  |  |  |  |  |  |  | Other countries outside of Europe | | | | | | | | | | |
| WORK ENVIRONMENT | | | | | | | | | | | | | | | | | | | | | | | | | | | | | | | | | | | | | | | | | | | | | | | | | | |
| **Job status/ Employment status** | | | | | | | | Employed | | | | | | | | | | | | Unemployed | | | | | | | | Retired | | | | | | Student, domestic | | | | | | | | | Permanent disable, other inactive status | | | | | | | |
| **Full-time or part-time job** | | | | Full-time | | | | | | | | | | | **If you are an employee, are you employed?** | | | | | | | | | Permanent work contract of unlimited duration | | | | **Kind of road** | | | | | Motorway | | | | | | **How do you go to work?** | | | | | | | | Car | | | |
|  |  |  |  | Part-time | | | | | | | | | | |  |  |  |  |  |  |  |  |  | Temporary work contract of limited duration | | | |  |  |  |  |  | Main road in town or urban area | | | | | |  |  |  |  |  |  |  |  | Bike/ foot | | | |
| **Self-employee or an employee** | | | | Self-employed | | | | | | | | | | | **How far do you work from a heavy traffic road (i.e. in town or countryside)** | | | | | | | | | Less than 100 m | | | |  |  |  |  |  | Main road outside town or urban area | | | | | |  |  |  |  |  |  |  |  | Bus/ train | | | |
|  |  |  |  | Employed | | | | | | | | | | |  |  |  |  |  |  |  |  |  | More than 500 m | | | |  |  |  |  |  | Other | | | | | |  |  |  |  |  |  |  |  | Other | | | |
| PRIVATE ENVIRONMENT | | | | | | | | | | | | | | | | | | | | | | | | | | | | | | | | | | | | | | | | | | | | | | | | | | |
| **Type of household** | | | | | | | | | One-person household | | | | | | | | | | | | **Has any building work been done in your home over the past three month**s? | | | | | | | Yes | | | | | | | **One or more pets** | | | | | | | | | Yes | | | | | | |
|  |  |  |  |  |  |  |  |  | Multi-person household | | | | | | | | | | | |  |  |  |  |  |  |  | No | | | | | | |  |  |  |  |  |  |  |  |  | No | | | | | | |
| **If multi-person household** | | | Community without kids less than 25 | | | | | | | | | | **When has the building you live in been built?** | | | | | | | | | | Less than 10 years ago | | | | | **How far do you live from a heavy traffic road?** | | | | Less than 100 m | | | | | | **Kind of road?** | | | | | | | | Motorway | | | | |
|  |  |  | Community with kids less than 25 | | | | | | | | | |  |  |  |  |  |  |  |  |  |  | Between 10 years and 30 years ago | | | | |  |  |  |  | More than 100 m and less than 500 m | | | | | |  |  |  |  |  |  |  |  | Main road in town or urban area | | | | |
|  |  |  | Other type | | | | | | | | | |  |  |  |  |  |  |  |  |  |  | Over 30 years ago | | | | |  |  |  |  | More than 500 m | | | | | |  |  |  |  |  |  |  |  | Main road outside town or urban area | | | | |
| **Do you have a garden?** | | | | | | | | | Yes | | | | | | | | | | | | **Pesticides using in your garden?** | | | | | | | Yes | | | | | | | **Pesticides using inside your home (herbicides, insecticides,** **fungicides, etc.)?** | | | | | | | | | Yes | | | | | | |
|  |  |  |  |  |  |  |  |  | No | | | | | | | | | | | |  |  |  |  |  |  |  | No | | | | | | |  |  |  |  |  |  |  |  |  | No | | | | | | |
| EDUCATION | | | | | | | | | | | | | | | | | | | | | | | | | | | | | | | | | | | | | | | | | | | | | | | | | | |
| **Education level** | | | | | | | | | | | | | | | | Primary | | | | | | | | | | | | | Secondary | | | | | | | | | | | Tertiary | | | | | | | | | | |
| GENERAL HEALTH | | | | | | | | | | | | | | | | | | | | | | | | | | | | | | | | | | | | | | | | | | | | | | | | | | |
| **Health status** | | | | | | | | | | | | | | | | Very good, good | | | | | | | | | | | | **Longstanding illness or health problems** | | | | | | | | | | | | Yes | | | | | | | | | | |
|  |  |  |  |  |  |  |  |  |  |  |  |  |  |  |  | Fair, bad, very bad | | | | | | | | | | | |  |  |  |  |  |  |  |  |  |  |  |  | No | | | | | | | | | | |
| **BMI (continuous)** | | | | | | | | | | | | | | | | **Age (continuous)** | | | | | | | | | | | | **Weight (continuous)** | | | | | | | | | | | | **Height (continuous)** | | | | | | | | | | |
| CHRONIC DISEASES, have you ever had (options to answer yes or no) | | | | | | | | | | | | | | | | | | | | | | | | | | | | | | | | | | | | | | | | | | | | | | | | | | |
| **Hyper-tension** | **Elevated blood chol-esterol** | | | | | | | | | | **Diabetes** | | | | | | | | **Coronary heart disease or angina pectoris** | | | | | | | **Myo-cardial infarction (heart attack)** | | **Stroke** | | | **Stomach ulcer** | | | | | **Cirrhosis of the liver, liver dys-function** | | | | | **Urinary incon-tinence** | | | | | | | | | **Kidney issues** |
| **Rheuma-toid arthritis** | **Arthrosis** | | | | | | | | | | **Osteo-porosis** | | | | | | | | **Cancer (malig-nant tumor, also including leukemia and lymph-oma)** | | | | | | | **Severe headache such as migraine** | | **Chronic anxiety** | | | **Depres-sion** | | | | | **Metabolic syndrome** | | | | | **Low Back disorder or other chronic back defect** | | | | | | | | | **Neck disorder or other chronic neck defect** |
| CHRONIC DISEASES, have you had in the last 12 months (options to answer yes or no) | | | | | | | | | | | | | | | | | | | | | | | | | | | | | | | | | | | | | | | | | | | | | | | | | | |
| **Hyper-tension** | **Elevated blood chol-esterol** | | | | | | | | | | **Diabetes** | | | | | | | | **Coronary heart disease or angina pectoris** | | | | | | | **Myo-cardial infarction (heart attack)** | | **Stroke** | | | **Stomach ulcer** | | | | | **Cirrhosis of the liver, liver dys-function** | | | | | **Urinary incon-tinence** | | | | | | | | | **Kidney issues** |
| **Rheuma-toid arthritis** | **Arthrosis** | | | | | | | | | | **Osteo-porosis** | | | | | | | | **Cancer (malig-nant tumor, also including leukemia and lymph-oma)** | | | | | | | **Severe headache such as migraine** | | **Chronic anxiety** | | | **Depres-sion** | | | | | **Metabolic syndrome** | | | | | **Low Back disorder or other chronic back defect** | | | | | | | | | **Neck disorder or other chronic neck defect** |
| PAIN | | | | | | | | | | | | | | | | | | | | | | | | | | | | | | | | | | | | | | | | | | | | | | | | | | |
| **Body pain (during the past 4 weeks)** | | | | | | | | | | | | | | | None | | | | | | | | | | | | | **During the past 4 weeks, how much did pain interfere with your normal work (including work outside the home and housework)?** | | | | | | | | | | | | Not at all | | | | | | | | | | |
|  |  |  |  |  |  |  |  |  |  |  |  |  |  |  | Mild to moderate | | | | | | | | | | | | |  |  |  |  |  |  |  |  |  |  |  |  | Little to moderate | | | | | | | | | | |
|  |  |  |  |  |  |  |  |  |  |  |  |  |  |  | Severe to very severe | | | | | | | | | | | | |  |  |  |  |  |  |  |  |  |  |  |  | Quite to extreme | | | | | | | | | | |
| MENTAL WELLBENIG (answer options yes or no) | | | | | | | | | | | | | | | | | | | | | | | | | | | | | | | | | | | | | | | | | | | | | | | | | | |
| **Feeling bad about yourself** | | | **Feeling down, depressed or hopeless** | | | | | | | | | | | **Trouble falling, staying asleep, sleeping to much** | | | | | | | | | | **Tired, little energy** | | | | **Poor appetite or overeating** | | | | | **Trouble concentrating on things** | | | | | | | **Moving or speaking slowly/ being fidgety or restless** | | | | | | | | | **Suicidal thoughts or hurting yourself** | |
| SLEEP | | | | | | | | | | | | | | | | | | | | | | | | | | | | | | | | | | | | | | | | | | | | | | | | | | |
| **Sleep hours every day** | | | | | | | | | | | | | | | | ≤ 6 h | | | | | | | | | | | | **Do you have problems with sleeping through the night** | | | | | | | | | | | | | | Yes | | | | | | | | |
|  |  |  |  |  |  |  |  |  |  |  |  |  |  |  |  | 7-8 h | | | | | | | | | | | |  |  |  |  |  |  |  |  |  |  |  |  |  |  | No | | | | | | | | |
|  |  |  |  |  |  |  |  |  |  |  |  |  |  |  |  | > 8 h | | | | | | | | | | | |  |  |  |  |  |  |  |  |  |  |  |  |  |  | Missing | | | | | | | | |
| **Sleep hours when working next day** | | | | | | | | | | | | | | | | ≤ 6 h | | | | | | | | | | | | **Do you snore?** | | | | | | | | | | | | | | Yes | | | | | | | | |
|  |  |  |  |  |  |  |  |  |  |  |  |  |  |  |  | 7-8 h | | | | | | | | | | | |  |  |  |  |  |  |  |  |  |  |  |  |  |  | No | | | | | | | | |
|  |  |  |  |  |  |  |  |  |  |  |  |  |  |  |  | > 8 h | | | | | | | | | | | |  |  |  |  |  |  |  |  |  |  |  |  |  |  | I don`t know | | | | | | | | |
| **Sleep and job status** | | | | | | | | | | | | | | | | ≤ 6 h and working | | | | | | | | | | | | **Do you take an afternoon nap or some kind of other nap during the day** | | | | | | | | | | | | | | Yes | | | | | | | | |
|  |  |  |  |  |  |  |  |  |  |  |  |  |  |  |  | > 6 h and working | | | | | | | | | | | |  |  |  |  |  |  |  |  |  |  |  |  |  |  | No | | | | | | | | |
|  |  |  |  |  |  |  |  |  |  |  |  |  |  |  |  | ≤ 6 h and not working | | | | | | | | | | | | **How often do you feel tired or fatigued after your sleep** | | | | | | | | | | | | | | Yes | | | | | | | | |
|  |  |  |  |  |  |  |  |  |  |  |  |  |  |  |  | > 6 h and not working | | | | | | | | | | | |  |  |  |  |  |  |  |  |  |  |  |  |  |  | No | | | | | | | | |
| HEALTHCARE MODULE - MEDICINE USE, (options to answer yes or no) | | | | | | | | | | | | | | | | | | | | | | | | | | | | | | | | | | | | | | | | | | | | | | | | | | |
| **Medicine use, that were described for you by a doctor (last two weeks)** | | | | | | | | | | | | | | | | | | | | | | | | | | | | | | | | | | | | | Yes | | | | | | | | No | | | | | |
| If yes, Medication was prescribed for… (options to answer yes or no) | | | | | | | | | | | | | | | | | | | | | | | | | | | | | | | | | | | | | | | | | | | | | | | | | | |
| **High blood pressure** | | | | | | | **Lowering the blood cholesterol level** | | | | | | | | | | | **Other cardiovascular disease, such as stroke and heart attack** | | | | | | | | | **Diabetes** | | | **Stomach troubles** | | | | | | | **Pain in the joints** | | | | | | | | **Pain in the neck or back** | | | | | |
| **Headache or migraine** | | | | | | **Depression** | | | | | | | | | | | | **Sleeping Tablets (last two weeks)** | | | | | | | | | | **Antibiotics (last two weeks)** | | | | | | | | | **Cancer** | | | | | | | | **Tension or anxiety** | | | | | |
| **Medicine use or herbal medicines or vitamins not described by a doctor (past two weeks)** | | | | | | | | | | | | | | | | | | | | | | | | | | | | | | | | | | | | | Yes | | | | | | | | No | | | | | |
| If yes, Medication was taken for… (options to answer yes or no) | | | | | | | | | | | | | | | | | | | | | | | | | | | | | | | | | | | | | | | | | | | | | | | | | | |
| **Pain in the joints** | | | | | | **Pain in the neck or back** | | | | | | | | | | | | **Headache or migraine** | | | | | | | | | | **Cold, flu or sore throat** | | | | | | | | | **Stomach troubles** | | | | | | | | **Vitamins, minerals or tonics** | | | | | |
| PREVENTIVE SERVICE | | | | | | | | | | | | | | | | | | | | | | | | | | | | | | | | | | | | | | | | | | | | | | | | | | |
| **Vaccination against flu (in your lifetime)** | | | | | | Yes | | | | | | | | | | | | **Blood pressure** | | | | | | | | | | Within the past 12 months | | | | | | | | | **Blood cholesterol** | | | | | | | | Within the past 12 months | | | | | |
|  |  |  |  |  |  | No | | | | | | | | | | | |  |  |  |  |  |  |  |  |  |  | Longer than 12 months | | | | | | | | |  |  |  |  |  |  |  |  | Longer than 12 months | | | | | |
| **Blood sugar** | | | | | | Within the past 12 months | | | | | | | | | | | | **Faecal occult blood test** | | | | | | | | | | Within the past 12 months | | | | | | | | | **Colonoscopy** | | | | | | | | Within the past 12 months | | | | | |
|  |  |  |  |  |  | Longer than 12 months | | | | | | | | | | | |  |  |  |  |  |  |  |  |  |  | 1 to less than 2 years | | | | | | | | |  |  |  |  |  |  |  |  | 1 to less than 5 years | | | | | |
| UNMET NEEDS FOR HEALTHCARE (options to answer yes, no or no need for healthcare) | | | | | | | | | | | | | | | | | | | | | | | | | | | | | | | | | | | | | | | | | | | | | | | | | | |
| **Absence from work due to personal health problems (last 12 months)?** | | | | | | | | | | | | | | | | | | | | | Yes | | | | | | | | | | | | | | No | | | | | | | | | | | | | | | |
| **The time needed to obtain an appointment was too long** | | **Distance or transportation problem** | | | | | | | | | | **Medical examination** | | | | | | | | | | | **Dental examination** | | | | | **Prescribed medicines** | | | | | **Eyeglasses or hearing aids** | | | | | | **Mental health care** | | | | | | | | | **Dietician** | | |
| HEALTH DETERMINANT MODULE - PHYSICAL ACTIVITY AND EXERCISE | | | | | | | | | | | | | | | | | | | | | | | | | | | | | | | | | | | | | | | | | | | | | | | | | | |
| **Fitness** | | | | | | | | | | Never | | | | | | | | | | | | **Work-related physical activity** | | | | | | Mostly physical active | | | | | | | **Total of physical activity** | | | | | | | | | Not PA | | | | | | |
|  |  |  |  |  |  |  |  |  |  | ≤ 3 h/week | | | | | | | | | | | |  |  |  |  |  |  | Not physical active | | | | | | |  |  |  |  |  |  |  |  |  | PA | | | | | | |
|  |  |  |  |  |  |  |  |  |  | > 3 h/week | | | | | | | | | | | |  |  |  |  |  |  | Not working | | | | | | |  |  |  |  |  |  |  |  |  |  |  |  |  |  |  |  |
| **How much time do you spend walking to get to and from places on atypical day** | | | | | | | | | | | | | | | | 10-29 minutes per day | | | | | | | | | | | | **How much time do you spend bicycling to get to and from places on atypical day?** | | | | | | | | | | | | 10-29 minutes per day | | | | | | | | | | |
|  |  |  |  |  |  |  |  |  |  |  |  |  |  |  |  | 30-59 minutes per day | | | | | | | | | | | |  |  |  |  |  |  |  |  |  |  |  |  | 30-59 minutes per day | | | | | | | | | | |
|  |  |  |  |  |  |  |  |  |  |  |  |  |  |  |  | 1 hour to less than 2 hours per day | | | | | | | | | | | |  |  |  |  |  |  |  |  |  |  |  |  | 1 hour to less than 2 hours per day | | | | | | | | | | |
|  |  |  |  |  |  |  |  |  |  |  |  |  |  |  |  | 2 hours to less than 3 hours per day | | | | | | | | | | | |  |  |  |  |  |  |  |  |  |  |  |  | 2 hours to less than 3 hours per day | | | | | | | | | | |
|  |  |  |  |  |  |  |  |  |  |  |  |  |  |  |  | 3 hours or more per day | | | | | | | | | | | |  |  |  |  |  |  |  |  |  |  |  |  | 3 hours or more per day | | | | | | | | | | |
|  |  |  |  |  |  |  |  |  |  |  |  |  |  |  |  | don`t know | | | | | | | | | | | |  |  |  |  |  |  |  |  |  |  |  |  | don`t know | | | | | | | | | | |
| NUTRITIONAL HABITS* | | | | | | | | | | | | | | | | | | | | | | | | | | | | | | | | | | | | | | | | | | | | | | | | | | |
| **Diet** | | | | | Standard | | | | | | | | | | | | **Consump-tion of fruits and vegetables** | | | | | | | | 0 portion per day | | | | **Looking at the nutritional information on food packaging** | | | | Yes | | | | | | | **Currently on a specific diet** | | | | | | | | | Yes | |
|  |  |  |  |  | Vegetarian | | | | | | | | | | | |  |  |  |  |  |  |  |  | 1-4 portions per day | | | |  |  |  |  | No | | | | | | |  |  |  |  |  |  |  |  |  | No | |
|  |  |  |  |  | Vegan | | | | | | | | | | | |  |  |  |  |  |  |  |  | 5 and more portions per day | | | | **influence of (159 question) of food purchases** | | | | Yes | | | | | | | **what type of sugar or sweetener do you use?** | | | | | | | | | Sugar (white, brown) | |
|  |  |  |  |  | Other | | | | | | | | | | | |  |  |  |  |  |  |  |  | No answer | | | |  |  |  |  | No | | | | | | |  |  |  |  |  |  |  |  |  | Others | |
| **Spices/ herbs use** | | | | | Yes, always | | | | | | | | | | | | **Salt and/or stock cubes, Aromat, Maggi use** | | | | | | | | Yes, salt only | | | | **Do you put salt in your food before eating** | | | | Yes, always | | | | | | | **Sugar use in tea or coffee or yogurt** | | | | | | | | | Yes, always | |
|  |  |  |  |  | Yes, from time to time | | | | | | | | | | | |  |  |  |  |  |  |  |  | Yes, salt and other flavorings | | | |  |  |  |  | Yes, from time to time | | | | | | |  |  |  |  |  |  |  |  |  | Yes, from time to time | |
|  |  |  |  |  | No, never | | | | | | | | | | | |  |  |  |  |  |  |  |  | No, I add nothing | | | |  |  |  |  | No, never | | | | | | |  |  |  |  |  |  |  |  |  | No, never | |
| **Organic foods** | | | | | Yes, always | | | | | | | | | | | | **How often do you eat barbecued food in the summer** | | | | | | | | Up to 6 times per week | | | | **Where do you usually eat at lunchtime** | | | | Restaurant | | | | | | | **Do you do the food shopping** | | | | | | | | | Yes, always | |
|  |  |  |  |  | Yes, from time to time | | | | | | | | | | | |  |  |  |  |  |  |  |  | Less than 6 times per week | | | |  |  |  |  | At home | | | | | | |  |  |  |  |  |  |  |  |  | Yes, from time to time | |
|  |  |  |  |  | No, never | | | | | | | | | | | |  |  |  |  |  |  |  |  | Never | | | |  |  |  |  | Other | | | | | | |  |  |  |  |  |  |  |  |  | No, never | |
| TOBACCO | | | | | | | | | | | | | | | | | | | | | | | | | | | | | | | | | | | | | | | | | | | | | | | | | | |
| **Smoking** | | | | | | | | | | | | | | | | Never | | | | | | | | | | | | **How often are you exposed to tobacco smoke indoors** | | | | | | | | | | | | | | Never or almost never | | | | | | | | |
|  |  |  |  |  |  |  |  |  |  |  |  |  |  |  |  | Current | | | | | | | | | | | |  |  |  |  |  |  |  |  |  |  |  |  |  |  | Less than 1 hour per day | | | | | | | | |
|  |  |  |  |  |  |  |  |  |  |  |  |  |  |  |  | Ex-Smokers | | | | | | | | | | | |  |  |  |  |  |  |  |  |  |  |  |  |  |  | 1 hour or more a day | | | | | | | | |
| ALCOHOL | | | | | | | | | | | | | | | | | | | | | | | | | | | | | | | | | | | | | | | | | | | | | | | | | | |
| **Alcohol consumption** | | | | | | No current drinkers | | | | | | | | | | | | **Ever felt bad or guilty about drinking** | | | | | | | | | | Yes | | | | | | | | | **Drinks first thing in the morning to steady your nerves or get rid of a hangover** | | | | | | | | Yes | | | | | |
|  |  |  |  |  |  | Current drinkers | | | | | | | | | | | |  |  |  |  |  |  |  |  |  |  | No | | | | | | | | |  |  |  |  |  |  |  |  | No | | | | | |
| ALLERGIES (options to answer yes or no) | | | | | | | | | | | | | | | | | | | | | | | | | | | | | | | | | | | | | | | | | | | | | | | | | | |
| **During your life, has ever one of the following diseases or health problems been diagnosed by a medical doctor?** | | | | | | | | | | | | | | | | | | | | | | | | | | | | **In the past 12 months, have you had any of the following diseases?** | | | | | | | | | | | | | | | | | | | | | | |
| **Eye inflammation** | | | | | | | | | | | | | | | | **Food allergy** | | | | | | | | | | | | **Eye inflammation** | | | | | | | | | | | | | | **Food allergy** | | | | | | | | |
| **Nasal allergies including hay fever** | | | | | | | | | | | | | | | | **Asthma (allergic asthma included)** | | | | | | | | | | | | **Nasal allergies including hay fever** | | | | | | | | | | | | | | **Asthma (allergic asthma included)** | | | | | | | | |
| **Eczema or any kind of skin allergy** | | | | | | | | | | | | | | | | **Allergy (at least one)** | | | | | | | | | | | | **Eczema or any kind of skin allergy** | | | | | | | | | | | | | | **Allergy (at least one)** | | | | | | | | |

# Questions asked in the EHES-LUX questionnaire and included in this study were defined as variables, indicated in bold in the table. *12 variables on regularity of food consumption on work and rest days.

**Table E4.** Sensitization levels to all allergens and extracts, separated into allergen groups for all sensitized participants (N=643), as visualized in Figure E2, A.

| IgE [kU_A_/L] | SENSITIZED PARTICIPANTS [%] | | | | | | | | | | | |
| --- | --- | --- | --- | --- | --- | --- | --- | --- | --- | --- | --- | --- |
|  | **Respiratory** | | | | | | | | | | | |
|  | Tree pollen | | Grass pollen | | Weed pollen | | Mites | | Dander | | Molds | |
| > 0.3 - < 1 | 12.1 | | 10.0 | | 7.3 | | 8.4 | | 6.5 | | 3.9 | |
| ≥ 1 - < 5 | 13.5 | | 13.8 | | 6.4 | | 11.2 | | 10.7 | | 4.5 | |
| ≥ 5 | 26.7 | | 28.0 | | 2.5 | | 20.7 | | 12.6 | | 4.8 | |
| Total sensitized | 52.4 | | 51.8 | | 16.2 | | 40.3 | | 29.9 | | 13.2 | |
| Total population | 20.6 | | 20.4 | | 6.6 | | 15.9 | | 11.9 | | 5.2 | |
|  | **Food** | | | | | | | | | | | |
|  | Legumes | Fruit | | Seafood | | Cereals | Vegetables | Egg/Milk | | Meat | | Spices |
| > 0.3 - < 1 | 17.6 | 11.7 | | 18.0 | | 17.3 | 10.4 | 3.4 | | 9.6 | | 10.4 |
| ≥ 1 - < 5 | 10.1 | 13.2 | | 8.2 | | 6.8 | 5.9 | 1.1 | | 5.6 | | 0.5 |
| ≥ 5 | 8.9 | 8.2 | | 2.6 | | 0.8 | 2.0 | 0.2 | | 1.2 | | 0.0 |
| Total sensitized | 36.5 | 33.1 | | 28.9 | | 24.9 | 18.4 | 4.7 | | 16.5 | | 10.9 |
| Total population | 14.3 | 13.2 | | 11.6 | | 9.3 | 7.6 | 0.7 | | 7.7 | | 2.4 |
|  | **Other** | | | | | | | | | | | |
|  | Venom | | | | | | Others | | | | | |
| > 0.3 - < 1 | 17.0 | | | | | | 4.0 | | | | | |
| ≥ 1 - < 5 | 12.0 | | | | | | 2.5 | | | | | |
| ≥ 5 | 5.4 | | | | | | 1.1 | | | | | |
| Total sensitized | 34.4 | | | | | | 7.6 | | | | | |
| Total population | 13.7 | | | | | | 3.2 | | | | | |

**Table E5.** Sensitization levels for allergens eliciting specific IgE responses higher 5 kU_A_/L, for all participants reaching that level of sensitization (N=346) as visualized in Figure 2.

| **TREE POLLEN** | | | | | | | | | | | | | | | | | | | | | | | | | | | | | | | | | | | | | | | | | | | | | |
| --- | --- | --- | --- | --- | --- | --- | --- | --- | --- | --- | --- | --- | --- | --- | --- | --- | --- | --- | --- | --- | --- | --- | --- | --- | --- | --- | --- | --- | --- | --- | --- | --- | --- | --- | --- | --- | --- | --- | --- | --- | --- | --- | --- | --- | --- |
|  | Aln g 1 | | | | | Aln g 4 | | | | | Fra e 1 | | | | | Fra e | | | | | Fag s 1 | | | | | Cor a 1.0103 | | | | | Cor a pollen | | | | | | Ole e 1 | | | | | Bet v 1 | | | |
| Participants sensitized | 46 | | | | | 6 | | | | | 84 | | | | | 59 | | | | | 92 | | | | | 100 | | | | | 50 | | | | | | 34 | | | | | 128 | | | |
| Participants sensitized [%] | 7.2 | | | | | 0.9 | | | | | 13.1 | | | | | 9.2 | | | | | 14.3 | | | | | 15.6 | | | | | 7.8 | | | | | | 5.3 | | | | | 19.9 | | | |
| Range [kU_A_/L] | 0.00 - 31.66 | | | | | 0.00 - 159.50 | | | | | 0.00 - 77.50 | | | | | 0.00 - 32.43 | | | | | 0.00 – 318.00 | | | | | 0.00 - 200.50 | | | | | 0.00 - 30.40 | | | | | | 0.00 - 29.33 | | | | | 0.00 - 452.50 | | | |
| Mean [kU_A_/L] | 1.35 | | | | | 0.74 | | | | | 3.73 | | | | | 2.09 | | | | | 4.84 | | | | | 4.92 | | | | | 1.60 | | | | | | 0.97 | | | | | 10.49 | | | |
| Mean kU_A_/L of sensitized participants | 10.20 | | | | | 42.90 | | | | | 15.43 | | | | | 12.34 | | | | | 18.29 | | | | | 17.13 | | | | | 11.16 | | | | | | 9.98 | | | | | 28.52 | | | |
| **GRASS POLLEN** | | | | | | | | | | | | | | | | | | | | | | | | | | | | | | | | | | | | | | | | | | | | | |
|  | Pas n | | | | Cyn d 1 | | | | | Cyn d | | | | | Lol p 1 | | Sec c pollen | | | | | Phl p 5.0101 | | | Phl p 1 | | | | | Phl p 6 | | | Phl p 2 | | | | | Phl p 7 | | | | | Phl p 12 | | |
| Participants sensitized | 19 | | | | 42 | | | | | 7 | | | | | 103 | | 50 | | | | | 132 | | | 133 | | | | | 93 | | | 72 | | | | | 6 | | | | | 6 | | |
| Participants sensitized [%] | 3.0 | | | | 6.5 | | | | | 1.1 | | | | | 16.0 | | 7.8 | | | | | 20.5 | | | 20.7 | | | | | 14.5 | | | 11.2 | | | | | 0.9 | | | | | 0.9 | | |
| Range [kU_A_/L] | 0.00 - 20.65 | | | | 0.00 - 27.31 | | | | | 0.00 - 17.80 | | | | | 0.00 - 34.45 | | 0.00 - 26.85 | | | | | 0.00 – 136.00 | | | 0.00 – 143.00 | | | | | 0.00 - 89.50 | | | 0.00 – 83.00 | | | | | 0.00 – 120.00 | | | | | 0.00 - 21.68 | | |
| Mean [kU_A_/L] | 0.49 | | | | 1.25 | | | | | 0.18 | | | | | 3.85 | | 1.68 | | | | | 7.84 | | | 6.24 | | | | | 4.26 | | | 2.98 | | | | | 0.71 | | | | | 0.20 | | |
| Mean kU_A_/L of sensitized participants | 8.96 | | | | 10.35 | | | | | 8.81 | | | | | 13.00 | | 11.69 | | | | | 20.67 | | | 16.32 | | | | | 15.95 | | | 14.40 | | | | | 41.25 | | | | | 11.69 | | |
| **DANDER** | | | | | | | | | | | | | | | | | | | | | | | | | | | | | | | | | | | | | | | | | | | | | |
|  | Fel d 1 | | | | | | Fel d 7 | | | | | Fel d 4 | | | | | | Can f 1 | | | | | Can f 4 | | | | | | Can f 6 | | | | | | Can f male urine | | | | | | Equ c 1 | | | | |
| Participants sensitized | 52 | | | | | | 16 | | | | | 10 | | | | | | 15 | | | | | 11 | | | | | | 6 | | | | | | 21 | | | | | | 10 | | | | |
| Participants sensitized [%] | 8.1 | | | | | | 2.5 | | | | | 1.6 | | | | | | 2.3 | | | | | 1.7 | | | | | | 0.9 | | | | | | 3.3 | | | | | | 1.6 | | | | |
| Range [kU_A_/L] | 0.00 – 53.00 | | | | | | 0.00 - 27.88 | | | | | 0.00 - 30.72 | | | | | | 0.00 - 29.45 | | | | | 0.00 - 26.44 | | | | | | 0.00 - 14.15 | | | | | | 0.00 - 34.92 | | | | | | 0.00 - 34.51 | | | | |
| Mean [kU_A_/L] | 2.73 | | | | | | 0.69 | | | | | 0.41 | | | | | | 0.53 | | | | | 0.44 | | | | | | 0.18 | | | | | | 0.81 | | | | | | 0.40 | | | | |
| Mean kU_A_/L of sensitized participants | 18.25 | | | | | | 14.98 | | | | | 14.36 | | | | | | 12.26 | | | | | 13.87 | | | | | | 10.16 | | | | | | 13.38 | | | | | | 13.94 | | | | |
| **MITES** | | | | | | | | | | | | | | | | | | | | | | | | | | | | | | | | | | | | | | | | | | | | | |
|  | Der f 2 | | Der f 1 | | | | | Der p 2 | | | | | Der p 1 | | | Der p 21 | | | Der p 23 | | | | | Der p 7 | | | | Der p 5 | | | | Der p 20 | | | | Bla g 9 | | | | Gly d 2 | | | | | Lep d 2 |
| Participants sensitized | 98 | | 35 | | | | | 98 | | | | | 55 | | | 17 | | | 32 | | | | | 19 | | | | 12 | | | | 8 | | | | 11 | | | | 11 | | | | | 51 |
| Participants sensitized [%] | 15.2 | | 5.4 | | | | | 15.2 | | | | | 8.6 | | | 2.6 | | | 5.0 | | | | | 3.0 | | | | 1.9 | | | | 1.2 | | | | 1.7 | | | | 1.7 | | | | | 7.9 |
| Range [kU_A_/L] | 0.00 - 132.80 | | 0.00 – 135.00 | | | | | 0.00 - 144.75 | | | | | 0.00 - 30.15 | | | 0.00 - 106.35 | | | 0.00 - 25.83 | | | | | 0.00 - 32.22 | | | | 0.00 - 25.68 | | | | 0.00 - 24.40 | | | | 0.00 - 31.70 | | | | 0.00 - 17.49 | | | | | 0.00 - 19.13 |
| Mean [kU_A_/L] | 5.79 | | 1.65 | | | | | 6.24 | | | | | 1.81 | | | 1.02 | | | 0.87 | | | | | 0.63 | | | | 0.44 | | | | 0.33 | | | | 0.48 | | | | 0.28 | | | | | 1.32 |
| Mean kU_A_/L of sensitized participants | 20.55 | | 16.36 | | | | | 22.15 | | | | | 11.45 | | | 20.79 | | | 9.44 | | | | | 11.50 | | | | 12.68 | | | | 14.20 | | | | 15.28 | | | | 8.96 | | | | | 9.03 |
| **MOULDS** | | | | **FRUITS** | | | | | | | | | | | | **LEGUMES** | | | | | | | | | | | **VEGETABLES** | | | | | | | | | | | | **VENOMS** | | | | | | |
|  | | Alt a 1 | | Mal d 1 | | | | | Cuc m 2 | | | | | Fra a 1+3 | | Ara h 8 | | | | Cor a 1.0401 | | | | Gly m 4 | | | Dau c 1 | | | | Dau c | | | Api g 1 | | | | | Ves v 5 | | | | | Ves v | |
| Participants sensitized | | 26 | | 33 | | | | | 7 | | | | | 32 | | 11 | | | | 50 | | | | 21 | | | 10 | | | | 7 | | | 11 | | | | | 28 | | | | | 9 | |
| Participants sensitized [%] | | 4.0 | | 5.1 | | | | | 1.1 | | | | | 5.0 | | 1.7 | | | | 7.8 | | | | 3.3 | | | 1.6 | | | | 1.1 | | | 1.7 | | | | | 4.4 | | | | | 1.4 | |
| Range [kU_A_/L] | | 0.00 - 32.66 | | 0.00 - 31.40 | | | | | 0.00 - 29.24 | | | | | 0.00 - 38.50 | | 0.00 - 18.70 | | | | 0.00 - 34.47 | | | | 0.00 - 35.51 | | | 0.00 - 29.73 | | | | 0.00 - 21.24 | | | 0.00 - 26.18 | | | | | 0.00 - 27.37 | | | | | 0.00 - 12.81 | |
| Mean [kU_A_/L] | | 0.84 | | 1.07 | | | | | 0.29 | | | | | 1.13 | | 0.28 | | | | 1.66 | | | | 0.74 | | | 0.30 | | | | 0.17 | | | 0.30 | | | | | 0.79 | | | | | 0.19 | |
| Mean kU_A_/L of sensitized participants | | 11.23 | | 11.27 | | | | | 14.33 | | | | | 12.26 | | 8.86 | | | | 11.58 | | | | 12.33 | | | 10.53 | | | | 8.48 | | | 9.48 | | | | | 9.79 | | | | | 7.17 | |

**Table E6.** Prevalence of sensitization against main groups of molecular allergens.

|  | **Sensitization** | | **Sensitization >5kU/L** | |
| --- | --- | --- | --- | --- |
| **Molecular group** | **Participants [no.]** | **Part of sensitized [%]** | **Participants [no.]** | **Part of sensitized [%]** |
| PR10 | 192 | 29.9 | 126 | 19.6 |
| NPC2 | 166 | 25.8 | 100 | 15.6 |
| Ole e1-like | 157 | 24.4 | 78 | 12.1 |
| Globulin 7/8S | 146 | 22.7 | 50 | 7.8 |
| Lipocalin | 72 | 11.2 | 29 | 4.5 |
| TLP | 44 | 6.8 | 2 | 0.3 |
| Profilin | 43 | 6.7 | 7 | 1.1 |
| Serum albumin | 39 | 6.1 | 18 | 2.8 |
| Albumin 2S | 38 | 5.9 | 0 | 0.0 |
| nsLTP | 33 | 5.1 | 3 | 0.5 |
| Tropomyosin | 21 | 3.3 | 3 | 0.5 |
| Polcalcin | 9 | 1.4 | 6 | 0.9 |
| Globulin 11S | 6 | 0.9 | 0 | 0.0 |

PR10 : Aln g 1,Api g 1,Ara h 8, Bet v 1, Cor a 1.0103, Dau c 1, Fag s 1, Gly m 4, Mal d 1; NPC2: Der f 2, Der p 2, Gly d 2, Lep d 2, Tyr p 2; OleE1Family: Che a 1, Fra e 1, Ole e 1, Pla l 1; Globulin 7/8S: Cor a 1.0401, Gly m 5, Jug r 1, Jug r 4, Pis v 3; Lipocalin: Arg r 1, Bla g 4, Can f 2, Can f 3, Can f FD1, Cav p1, Fel d 4, Del d 7, Mus m 1, Ory c 1, Ory c 2, Phod s 1; TLP: Act d 2, Mal d 2; Profilin: Bet v 2, Cuc m 2, Hev b 8, Mer a 1, Phl p 12, Pho d 2; Serum Albumin : Bos d 5, Can f 1, Fel d 2, Gal d 2; Albumin 2S: Ana o 3, Ber e 1, Cor a 12, Fag e 2, Gly m 8, Mac i 2S Albumin, Pap s 2S Albumin, Pis v 1, Ses I 1, Sin a 1; nsLTP: Act d 10, Api g 2, Api g 6, Art v 3, Can s 3, Cor a 14, Jug r 2, Mal d 3, Ole e 7, Par j 2, Pla a 3, Pru p 3, Sola l 6, Tri a aA TI, Vit v 1, Zea m 14; Tropomyosin: Ani s 3, Blo t 10, Pen m 1, Per a 7; Polcalcin: Aln g 4, Phl p 7; Globulin 11S: Ana o 2, Cor a 8, Gly m 6, Jug r 3, Pis v 2.

**Table E7.** Co-Sensitization levels to one or more allergen group, for all sensitized participants (N=643) divided by age group, as visualized in Figure 3, B.

| **SENSITIZATION PATTERN** | **25-34** | **35-44** | **45-54** | **55-65** | **25-34 vs**  **55-65** |
| --- | --- | --- | --- | --- | --- |
|  | Percentage [%] | | | | P-value |
| Mono-Sensitization to 1 source group | 10.6 | 27.3 | 18.1 | 49.5 | 9.855e-11 |
| Co-Sensitization to 2 source groups | 15.9 | 13.6 | 20.7 | 9.7 | 0.2304 |
| Co-Sensitization to 3 source groups | 9.8 | 9.1 | 16.5 | 10.7 | 1.00 |
| Co-Sensitization to 4-9 source groups | 45.5 | 40.0 | 43.6 | 28.2 | 0.009962 |
| Poly-Sensitization to ≥10 source groups | 18.2 | 10.0 | 1.1 | 1.9 | 0.0001927 |

**Table E8.** Spatial distribution of allergy ~~reports~~ diagnosis in the group of participants diagnosed with allergy & being sensitized, as well as the average population in 2013-2015, grouped by Luxembourg canton, as shown in Figure E3.

| **Canton number**# | **Canton** | **Diagnosed & sensitized**  **group [no.]** * | **Part of whole**  **cohort [%]** | **Diagnosed allergies [no.]** * | | | | | **Average population 2013-2015** |
| --- | --- | --- | --- | --- | --- | --- | --- | --- | --- |
|  |  |  |  | **Eye** | **Nasal** | **Skin** | **Food** | **Asthma** |  |
| **1** | Esch-sur-Alzette | 139 | 33.3 | 70 | 84 | 65 | 38 | 30 | 161107.0 |
| **2** | Remich | 21 | 33.9 | 8 | 16 | 11 | 6 | 4 | 20190.3 |
| **3** | Capellen | 45 | 31.7 | 23 | 33 | 22 | 10 | 11 | 43583.7 |
| **4** | Luxembourg | 152 | 34.4 | 77 | 107 | 66 | 39 | 31 | 167331.7 |
| **5** | Grevenmacher | 32 | 31.4 | 21 | 25 | 19 | 10 | 10 | 27309.0 |
| **6** | Redange | 10 | 22.2 | 5 | 7 | 4 | 4 | 1 | 17075.0 |
| **7** | Mersch | 29 | 30.2 | 11 | 18 | 14 | 9 | 5 | 29228.0 |
| **8** | Echternach | 16 | 26.7 | 4 | 12 | 6 | 4 | 1 | 17493.7 |
| **9** | Diekirch | 18 | 26.9 | 5 | 8 | 8 | 3 | 2 | 30413.0 |
| **10** | Wiltz | 10 | 27.0 | 6 | 6 | 3 | 2 | 3 | 14969.7 |
| **11** | Vianden | 3 | 15.8 | 1 | 2 | 2 | 0 | 1 | 4730.0 |
| **12** | Clervaux | 8 | 22.2 | 2 | 4 | 4 | 3 | 1 | 16461.3 |

# Numbering as shown in Figure E3. *participant numbers.

**Table E9**. Maximum of 15 top most stratifying allergens/extracts defined by the unsupervised clustering (Figure 4). Indicated are the allergens/extracts most different between the cluster and all other clusters.

| **Allergen/extract** | **Average log2 fold change** | **Adjusted P value** |
| --- | --- | --- |
| **CLUSTER 0** | | |
| Cor a 1.0401 | 4.4665 | 2.21E 100 |
| Cor a 1.0103 | 4.3997 | 2.11E 105 |
| Cor a pollen | 4.369258 | 1.58E 106 |
| Fag s 1 | 3.973092 | 3.52E 104 |
| Fra a1 3 | 3.958996 | 2.43E 78 |
| Aln g 1 | 3.832475 | 7.80E 94 |
| Gly m 4 | 3.591662 | 4.05E 58 |
| Bet v 1 | 3.460648 | 2.10E 98 |
| Dau c 1 | 3.104504 | 2.03E 38 |
| Ara h 8 | 2.981179 | 1.02E 51 |
| Mal d 1 | 2.945903 | 2.75E 102 |
| Api g 1 | 2.852659 | 4.23E 48 |
| Dau c | 2.492918 | 1.24E 32 |
| Ole e 1 | 0.932686 | 2.71E 12 |
| Fra e 1 | 0.652761 | 3.36E 14 |
| **CLUSTER 1** | | |
| Ves v 5 | 4.033284 | 2.05E 11 |
| Ves v | 3.227372 | 0.019641 |
| **CLUSTER 2** | | |
| Phl p5.0101 | 3.418631 | 2.07E 38 |
| Lol p 1 | 2.998777 | 8.13E 42 |
| Phl p 1 | 2.983481 | 3.16E 48 |
| Cyn d 1 | 2.79484 | 6.47E 18 |
| Sec c pollen | 2.710857 | 4.75E 21 |
| Phl p 6 | 2.694865 | 1.01E 16 |
| Cyn d | 2.549814 | 4.41E 10 |
| Phl p 2 | 2.382426 | 2.33E 10 |
| Pas n | 1.727472 | 0.001344 |
| **CLUSTER 3** | | |
| Der p 7 | 4.214294 | 2.33E 26 |
| Der p 2 | 3.980452 | 1.50E 64 |
| Der f 2 | 3.911281 | 1.09E 63 |
| Lep d 2 | 3.859411 | 2.31E 53 |
| Der p 1 | 3.779594 | 1.36E 45 |
| Der f 1 | 3.52054 | 3.59E 36 |
| Gly d 2 | 2.856033 | 1.60E 14 |
| Der p 23 | 2.06695 | 5.46E 29 |
| **CLUSTER 4** | | |
| Fra e | 3.187483 | 0.002043 |
| Fra e 1 | 3.11158 | 0.001388 |
| **CLUSTER 5** | | |
| Che q | 5.931344 | 5.68E 60 |
| Pop n | 5.58759 | 2.89E 57 |
| Ail a | 5.046517 | 9.43E 57 |
| Sec c flour | 5.009807 | 1.67E 55 |
| Pha v | 4.96703 | 9.77E 50 |
| Cup s | 4.740806 | 2.63E 49 |
| Cuc p | 4.261835 | 1.21E 21 |
| Cry j 1 | 4.233588 | 4.82E 42 |
| Ulm c | 3.591266 | 1.13E 32 |
| All c | 2.642191 | 2.85E 15 |
| Phr c | 2.53252 | 0.328065 |
| **CLUSTER 6** | | |
| Fel d 4 | 5.521779 | 2.72E 65 |
| Can f 1 | 5.126579 | 8.57E 40 |
| Fel d 7 | 5.110139 | 1.15E 49 |
| Can f 6 | 4.840764 | 8.34E 39 |
| Can f 3 | 4.822497 | 9.59E 34 |
| Equ c 1 | 4.599678 | 8.81E 32 |
| Equ c 3 | 4.574564 | 9.59E 34 |
| Fel d 2 | 4.551901 | 1.37E 40 |
| Can f 4 | 4.543063 | 6.58E 13 |
| Can f 2 | 4.291591 | 7.59E 38 |
| Sus d 1 | 3.657481 | 7.32E 30 |
| Can f 1 | 3.170097 | 4.58E 20 |
| Fel d 1 | 2.403586 | 1.96E 14 |
| Can f male urine | 0.922033 | 0.000142 |

**Table E10:** Significant differences between IgE cluster 0 (N=244) and all non-sensitized (A.) and otherwise sensitized (B.) participants (Figure 5).

| **A.) Comparison of IgE cluster 0 with non-sensitized participants** | | | | | | |
| --- | --- | --- | --- | --- | --- | --- |
| **Variable** | **Cluster 0** | **Cluster 0 [%]** | **Non-sensitized** | **Non-sensitized [%]** | **P value*** | **P value after BH correction** |
| Have you ever had any allergy? | | | | | | |
| No | 26 | 16.6 | 713 | 80.5 | < 2.2e 16 | 1.3e 15 |
| Yes | 131 | 83.4 | 171 | 19.3 |  |  |
| Have you ever had an eye allergy? | | | | | | |
| No | 71 | 45.2 | 846 | 95.5 | < 2.2e 16 | 1.3e 15 |
| Yes | 86 | 54.8 | 39 | 4.4 |  |  |
| Have you ever had food allergy? | | | | | | |
| No | 113 | 72.0 | 871 | 98.3 | < 2.2e 16 | 1.3e 15 |
| Yes | 44 | 28.0 | 14 | 1.6 |  |  |
| Have you ever had nasal allergy? | | | | | | |
| No | 39 | 24.8 | 824 | 93.0 | < 2.2e 16 | 1.3e 15 |
| Yes | 118 | 75.2 | 61 | 6.9 |  |  |
| Have you ever had asthma? | | | | | | |
| No | 119 | 75.8 | 824 | 93.0 | < 2.2e 16 | 1.3e 15 |
| Yes | 38 | 24.2 | 61 | 6.9 |  |  |
| Have you ever had skin allergy? | | | | | | |
| No | 108 | 68.8 | 786 | 88.7 | 8.05e 11 | 3.9e 10 |
| Yes | 49 | 31.2 | 99 | 11.2 |  |  |
| How old are you? | | | | | | |
| Mean age | 42.4 | | 45.7 | | 7.979e 05 | 3.4e 04 |
| Are you currently/have you ever been a smoker? | | | | | | |
| Ex smoker | 30 | 19.1 | 202 | 22.8 | 0.005493 | 0.01991 |
| No, never smoked | 104 | 66.2 | 469 | 52.9 |  |  |
| Yes, current smoker | 23 | 14.6 | 214 | 24.2 |  |  |
| Are you currently employed? | | | | | | |
| Employed | 135 | 86.0 | 662 | 74.7 | 0.008654 | 0.02194 |
| Other | 20 | 12.7 | 186 | 21.0 |  |  |
| Unemployed | 2 | 1.3 | 38 | 4.3 |  |  |
| In the last 12 months, have you needed health care but did not get it timely because the time needed to obtain an appointment was too long? | | | | | | |
| Yes | 32 | 20.4 | 125 | 14.1 | 0.009237 | 0.02679 |
| No | 81 | 51.6 | 569 | 64.2 |  |  |
| No need for healthcare | 44 | 28.0 | 191 | 21.6 |  |  |
| How is your overall health currently? | | | | | | |
| Bad/very bad | 5 | 3.2 | 45 | 5.1 | 0.02112 | 0.05568 |
| Fair | 20 | 12.7 | 188 | 21.2 |  |  |
| Very good/good | 132 | 84.1 | 653 | 73.7 |  |  |
| In the last 12 months, have you been feeling down, depressed or hopeless? | | | | | | |
| No | 129 | 82.2 | 649 | 73.3 | 0.02474 | 0.05979 |
| Yes | 28 | 17.8 | 236 | 26.6 |  |  |
| In the last 12 months, where you absent from work due to health problems? | | | | | | |
| Yes | 69 | 43.9 | 330 | 37.2 | 0.0272 | 0.06068 |
| No | 69 | 43.9 | 369 | 41.6 |  |  |
| I don’t work | 19 | 12.1 | 187 | 21.1 |  |  |
| **B.) Comparison of IgE cluster 0 with OTHER sensitized participants** | | | | | | |
| **Variable** | **Cluster 0** | **Cluster 0 [%]** | **Other sensitized** | **Other sensitized [%]** | **P value*** | **P value after BH correction** |
| Have you ever had nasal allergy? | | | | | | |
| No | 39 | 24.8 | 280 | 57.6 | 1.81e 12 | 5.25e 11 |
| Yes | 118 | 75.2 | 206 | 42.4 |  |  |
| Have you ever had an eye allergy? | | | | | | |
| No | 71 | 45.2 | 338 | 69.5 | 6.23e 08 | 9.04e 07 |
| Yes | 86 | 54.8 | 148 | 30.5 |  |  |
| Have you ever had asthma? | | | | | | |
| No | 119 | 75.8 | 421 | 86.6 | 0.003574 | 0.03455 |
| Yes | 38 | 24.2 | 65 | 13.4 |  |  |
| Have you ever had food allergy? | | | | | | |
| No | 113 | 72.0 | 401 | 82.5 | 0.005936 | 0.04304 |
| Yes | 44 | 28.0 | 85 | 17.5 |  |  |
| Have you ever had any allergy? | | | | | | |
| No | 26 | 16.6 | 132 | 27.2 | 0.01001 | 0.05806 |
| Yes | 131 | 83.4 | 354 | 72.8 |  |  |
| In the last 12 months, have you been feeling down, depressed or hopeless? | | | | | | |
| No | 129 | 82.2 | 348 | 71.6 | 0.01277 | 0.06172 |
| Yes | 28 | 17.8 | 137 | 28.2 |  |  |
| In the last 4 weeks, have you had any body pain? | | | | | | |
| None | 72 | 45.9 | 163 | 33.5 | 0.01818 | 0.06982 |
| Mild moderate | 77 | 49.0 | 284 | 58.4 |  |  |
| Severe very severe | 8 | 5.1 | 38 | 7.8 |  |  |
| Are you currently/have you ever been a smoker? | | | | | | |
| Ex smoker | 30 | 19.1 | 111 | 22.8 | 0.01926 | 0.06982 |
| No, never smoked | 104 | 66.2 | 262 | 53.9 |  |  |
| Yes, current smoker | 23 | 14.6 | 112 | 23.0 |  |  |
| How old are you? | | | | | | |
| Mean age | 42.4 | 44.2 | 0.03419 | 0.1102 |  |  |
| Are you currently employed? | | | | | | |
| Employed | 135 | 86.0 | 377 | 77.6 | 0.03927 | 0.1139 |
| Other | 20 | 12.7 | 86 | 17.7 |  |  |
| Unemployed | 2 | 1.3 | 23 | 4.7 |  |  |

BH, Benjamini-Hochberg correction, p-value was calculated with chi square- (categorical) or t-Test (continuous) statistical testing.
